# Widespread Occurrence of Dosage Compensation in *Candida albicans*

**DOI:** 10.1371/journal.pone.0010856

**Published:** 2010-06-11

**Authors:** Anatoliy Kravets, Hong Qin, Ausaf Ahmad, Gabor Bethlendy, Qinshan Gao, Elena Rustchenko

**Affiliations:** 1 Department of Biochemistry and Biophysics, University of Rochester Medical Center, Rochester, New York, United States of America; 2 Department of Biology, Spelman College, Atlanta, Georgia, United States of America; 3 Roche Diagnostics Corporation, Indianapolis, Indiana, United States of America; 4 Department of Microbiology, Mount Sinai School of Medicine, New York, New York, United States of America; Texas A&M University, United States of America

## Abstract

The important human pathogen *Candida albicans* possesses an unusual form of gene regulation, in which the copy number of an entire specific chromosome or a large portion of a specific chromosome changes in response to a specific adverse environment, thus, insuring survival. In the absence of the adverse environment, the altered portion of the genome can be restored to its normal condition. One major question is how *C*. *albicans* copes with gene imbalance arising by transitory aneuploid states. Here, we compared transcriptomes from cells with either two copies or one copy of chromosome 5 (Ch5) in, respectively, a diploid strain 3153A and its representative derivative Sor55. Statistical analyses revealed that at least 40% of transcripts from the monosomic Ch5 are fully compensated to a disomic level, thus, indicating the existence of a genome-wide mechanism maintaining cellular homeostasis. Only approximately 15% of transcripts were diminished twofold in accordance with what would be expected for Ch5 monosomy. Another minor portion of approximately 6% of transcripts, unexpectedly, increased up to twofold and higher than the disomic level, demonstrating indirect control by monosomy. Array comparative genome hybridization revealed that only few out of approximately 500 genes on the monosomic Ch5b were duplicated, thus, not causing a global up regulation. Dosage compensation was confirmed with several representative genes from another monosomic Ch5a in the mutant Sor60. We suggest that *C. albicans*'s unusual regulation of gene expression by the loss and gain of entire chromosomes is coupled with widespread compensation of gene dosage at the transcriptional level.

## Introduction

*Candida albicans* is a unicellular fungus that is a benign inhabitant of the mucosal surfaces of gastrointestinal tract in approximately two thirds of the healthy human population. In some healthy individuals, *C. albicans* causes merely superficial mucosal infections; however, in hospitals, *C. albicans* has emerged as an important pathogen of immunocompromized patients and is associated with significant morbidity, mortality, and high health-care costs.

*C. albicans* is an obligate diploid organism with 8 pairs of chromosomes, whose unusual instability has been systematically investigated by us and others [1]. Throughout years, we have accumulated evidence that *C. albicans* generates aneuploidies as a response to environmental stresses [1]–[3], despite aneuploidy having detrimental effects on cell division and growth similar to other organisms [4], [5]. Moreover, we have demonstrated that reversible loss or gain of specific chromosomes, or large portions of specific chromosomes, each occurring in a specific adverse environment, constitute unusual gene regulatory systems that allow *C. albicans* survival [1], [2]. In the best-studied case, adaptation to the toxic sugar sorbose, the major mechanism conferring survival is the loss of an entire chromosome 5a (Ch5a) or an entire chromosome 5b (Ch5b).

Here, we address an intriguing question of how *C. albicans* handles gene disbalance due to aneuploid chromosome by analyzing the expression of approximately 500 genes on the monosomic Ch5b. We found that at least 40% of transcripts were fully compensated to the disomic level, in contrast to another fungus *Saccharomyces cerevisiae*. This finding was supported with the analysis of limited number of genes from the alternative Ch5a. Using array comparative genome hybridization (aCGH), we demonstrated that widespread dosage compensation was not caused by gene duplication. This implies that epigenetic events may be responsible, similarly to compensatory mechanisms in higher eukaryotes, as elaborated in the Discussion.

## Results

### Evidence by pulsed-field gel electrophoresis (PFGE) that the mutant Sor55 is monosomic for Ch5b

Electrophoretic karyotype [6] of the mutant Sor55, which we analyzed here with expression or CGH arrays, was previously reported [7]. Sor55 derived from the laboratory strain 3153A by exposure to toxic sorbose, which caused the loss of Ch5a. We have previously established that the loss of an entire Ch5a is a predominant mechanism for adaptation to toxic sorbose [1], [2]. In this respect, Sor55 is a representative mutant.

**Figure 1:**
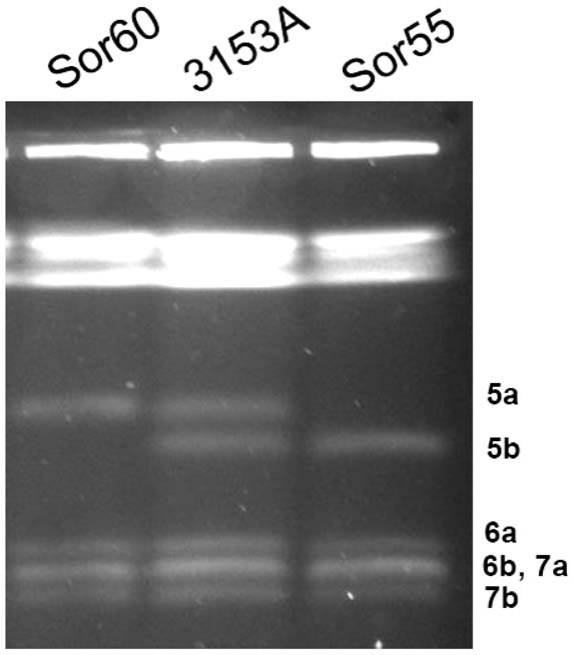
Evidence of Ch5 monosomy in the mutant Sor55 or Sor60. Electrophoretic karyotype is prepared using a contour-clamped homogeneous electric field apparatus (Bio-Rad Laboratories, Hercules, CA). Shown are precisely separated smaller Ch5, Ch6, and Ch7 of the parental strain 3153A, as well as of its derivatives Sor55 and Sor60. Homologous chromosomes are designated a or b. Under this running condition [3] that is optimizing smaller chromosomes, the longer chromosomes are compressed on the top of a gel. Separation of longer chromosomes is not presented. Note similar brightness of Ch5a or Ch5b in all strains (confirmation with densitometry is not presented). In contrast, a band containing co-migrating Ch6b and Ch7a is stained approximately twice stronger.

In order to assure that we are analyzing the transcriptom from cells with monosomic, but not with spontaneously duplicated Ch5b [7], we confirmed the electro-karyotype of Sor55. When cells of 3153A and Sor55 were grown to extract RNA for expression microarrays, an aliquot of cells was used to obtain native chromosomes for precise separation with PFGE [3] (Materials and Methods). As expected, Sor55 contained Ch5b, but lacked Ch5a, Fig. 1. The retained Ch5b was not duplicated, as determined by visual examination and densitometry [3]. In addition, consistently, PCR analysis showed that only *MTL*α*1* on Ch5b, but not *MTL* **a** *1* or *MTL* **a** *2* on Ch5a, could be amplified from genomic DNA of Sor55 with the appropriate primers [Table S1 of supporting information (SI)]. As expected, all three genes, *MTL*α1, *MTL* **a**1 and *MTL* **a**2, could be amplified from 3153A. Also, monosomy of Ch5 has been reported to up regulate the *SOU1* gene on Ch4 that is responsible for the growth on sorbose [7], [8]. As expected, *SOU1* in Sor55 was up regulated, as estimated by DNA microarray (Table S2) and Northern blot analysis (data not shown). We are, thus, confident that the Sor55 cells were monosomic for Ch5b at the time of transcriptom examination.

### Statistical analysis of the DNA microarray results reveals significantly diminished expression profile of monosomic Ch5b

Genome-wide transcription profiles were determined with our custom microarrays (Materials and Methods) for the Sor55 mutant, monosomic for Ch5b, and for its parental strain 3153A, disomic for Ch5. ANOVA (analysis of variance) showed that the influence of GC content of probes is negligible on expression changes in Sor55, as compared to 3153A. The expression change of each gene was estimated by either mean or median of the ratio Sor55/3153A. The p-value was estimated by pairwise t-test for base-2-log transformed expression signals for all genes along each chromosome. The data for each chip were normalized by assuming that the total intensity of all non-Ch5 genes remains the same. In our microarrays, Ch5 is represented by a total of 507 ORFs, of which 470 are expressed above the background noise in both 3153A and Sor55 (Material and Methods). The data in Table 1 show that monosomic Ch5b has, on average, a diminished expression profile of approximately 15–17%, as estimated by either mean or median of the ratios Sor55/3153A. The results are very similar when the expression data were normalized by the total intensity of all genes including Ch5 genes (data not shown). Chromosomes other than Ch5b did not have a decrease of overall expression. A detailed analysis of the genome-wide changes in expression will be presented elsewhere.

**Table 1:** Average change of gene expressions by chromosomes.

	Sor55/3153A			
Ch	Mean	Median	p-value	Gene number^a^
1	1.05	1.03	2.7×10^−6^	1346
R	1.04	1.03	0.0026	966
2	1.04	1.02	0.0011	987
3	1.04	1.00	0.019	745
4	1.14	1.10	1.2×10^−28^	661
5	0.86	0.85	1.3×10^−45^	507
6	1.03	1.02	0.31	431
7	1.12	1.06	4.4×10^−12^	406

a Genes with their expression within the background noise are excluded from calculating mean, median, and p-value, but not from the number of the genes on each chromosome. ChR refers to the chromosome containing a cluster of tandemly repeated rDNA units [9].

### Statistical analysis of the DNA microarray results reveals that expression of many genes on Ch5b are compensated to the diploid level, while expression of some other genes decreased or increased twofold or more

**Figure 2:**
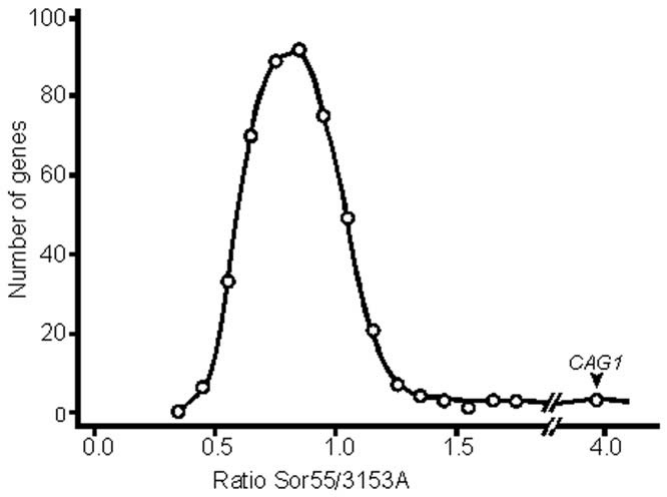
Distribution of expression ratios Sor55/3153A of Ch5b genes. A total of 470 genes with the expression above the background noise in our arrays are included. Expression ratio for each gene is calculated as the mean ratio Sor55/3153A. Expression ratios are binned along the abscissa. Each bin is 0.1. The number of genes in corresponding bins is presented along the ordinate.

The expression ratios Sor55/3153A of 470 Ch5b genes that are above the background noise (see above), comprise an approximately normal distribution covering a wide continuous range of values between more than twofold decrease to more than twofold increase, Fig. 2. This distribution was practically identical to the distribution that included all 507 Ch5 genes (data not shown). The values of expression ratios for all Ch5b genes can be accessed in SI, Table S2. A total of 186, or 40%, of the 470 genes had their ratios within the range from 0.9 to 1.1, which we considered the same as the disomic level. Of the remaining 284 genes 75 had statistically significant changes in expression, as determined with a p-value = 0.05 cutoff. The distribution of the expression ratios of those 75 genes covered the same wide range, as the distribution of all 470 genes (data not shown). We, thus, assumed that expression changes with no statistical significance may have biological significance and could become statistically significant if, for example, six chips instead of three were used for the arrays.

### Validation of the DNA microarray data for the expression of 38 genes on monosomic Ch5b

Northern blot and semi-quantitative RT-PCR analyses (Materials and Methods) were initially used for validation of the microarray data with two control genes not on Ch5b, *EMP24* (orf19.6293) on ChR (the chromosome containing rRNA genes) and orf19.2087 on Ch2. Both approaches confirmed the results obtained with the microarrays, showing no change of the expression ratios Sor55/3153A in these control genes (data not shown). We next choose at random 38 genes on monosomic Ch5b with various Sor55/3153A ratios and validated the ratios using two alternative approaches, as follows: 9 genes with both Northern blot and semi-quantitative RT-PCR analyses; 9 genes with only Northern blot; and 19 genes with only semi-quantitative RT-PCR analysis. Although, the values obtained with these two procedures agreed with each other and with those obtained with the microarray procedure, Northern blot analyses showed a larger variability with lower expressed genes and are, thus, considered less accurate. For this reason, we present in Table 2 the validation by RT-PCR for 21 representative genes. Validation by RT-PCR with all 30 genes or by Northern blot analyses with 21 genes is presented in supporting information, Tables S3 and S4, respectively. Expression changes of only two genes showed disagreement between microarray and RT-PCR methods, Table S3.

**Table 2:** RT-PCR validation^a^ of the array expression values with 21 genes^b^ on Ch5b in Sor55^c^.

		Sor55/3153A, Ch5b		Sor60/3153A, Ch5a
Expression on Ch5b	Gene	Arrays	RT-PCR	RT-PCR
4-fold up	*CAG1*	3.9	3.9; 4.0; 4.2	5.0; 5.1
2-fold up	*PGA37*	2.1	2.0; 2.0; 2.7	
	*GAP5*	1.7	1.4; 1.5; 1.8	
	*CAR1*	1.6	1.3; 1.8; 1.8	
	*GAP1*	1.6	1.4; 1.6; 1.7	
Disomic level	*HIS1*	1.0	1.0; 1.0; 1.0; 1.1	1.1; 1.1
	*UBC13*	1.0	1.0; 1.0; 1.0	
	*ACH1*	1.0	1.0; 1.0; 1.0; 1.1	
	*MDJ1*	1.0	1.0; 1.0; 1.0	
	*GLR1*	1.0	0.8; 0.9; 0.9; 1.0; 1.1	
	*PRE1*	1.0	0.8; 0.9; 0.9; 0.9	
	RPO26	1.0	0.9; 0.9; 1.0	
	orf19.4248	1.0	0.8; 0.9; 1.0	
	orf19.4349	1.0	0.8; 0.9; 1.0	
	*CTA24* d	1.0	1.0; 1.0; 1.0; 1.2	
Monosomic level	*SEC14*	0.5	0.5; 0.5; 0.5	
	*THS1*	0.5	0.5; 0.6; 0.7	
	orf19.3216	0.5	0.5; 0.6; 0.6	
	*PUT1*	0.5	0.3; 0.5; 0.6	0.4; 0.4
	*URA4*	0.5	0.5; 0.6; 0.6	
	*GDS1*	0.5	0.2; 0.5; 0.6	

a See Fig. S1 for examples of semi-quantitative RT-PCR gels.

b See Table S3 for all 30 genes, Table S1 for gene systematic names. For Northern blot analyses see the Results section and Table S4.

c Note that also shown are RT-PCR estimates of the expression level of several corresponding genes on Ch5a in Sor60.

d Note that *CTA24* is duplicated on Ch5b in Sor55, Table S2 and legend to Fig. 5, and is serving here, as a control for the expression from two copies of gene. The other genes have aCGH ratios Sor55/3153A ranging from 0.51 to 0.64 with two ratios equaling to 0.65 and 0.68. Most probably, all those genes possess a single copy.

Expression changes were calculated, as mean ratios Sor55/3153A for Ch5b or Sor60/3153A for Ch5a. Data were obtained from independent experiments.

The validation with two independent methods, Northern blot and RT-PCR, thus, supports the array results.

### Genes with approximately twofold change in expression, as compared to the diploid level, are scattered along the entire Ch5b

**Figure 3:**
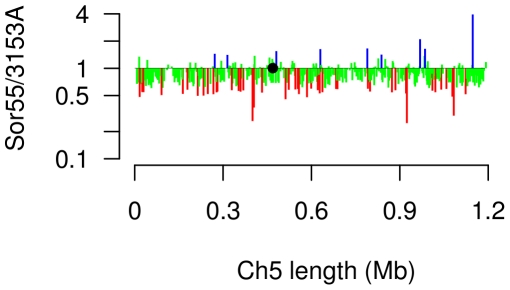
Distribution of expression ratios Sor55/3153A of Ch5b genes along Ch5. The mean expression ratio for each gene is plotted along the ordinate at the corresponding Ch5 position on the abscissa. Twofold or more diminished expressions are in red; twofold or more increased expressions are in blue. The remaining partially or fully compensated expressions are in green. For more detail see legend of Fig. 2. A black circle indicates the position of the centromere.

We prepared the distribution of expression changes along Ch5b by plotting the expression ratios Sor55/3153A of each gene to its position on Ch5b, Fig. 3. The genes with twofold or more decrease or increase, as compared to the diploid level, are in red and blue, respectively. The remaining genes are in green. It is obvious that two-fold down regulated or excessively up regulated genes are randomly located along the entire length of Ch5b with no clustering.

### DNA profiling of the monosomic Ch5b by aCGH

**Figure 4:**
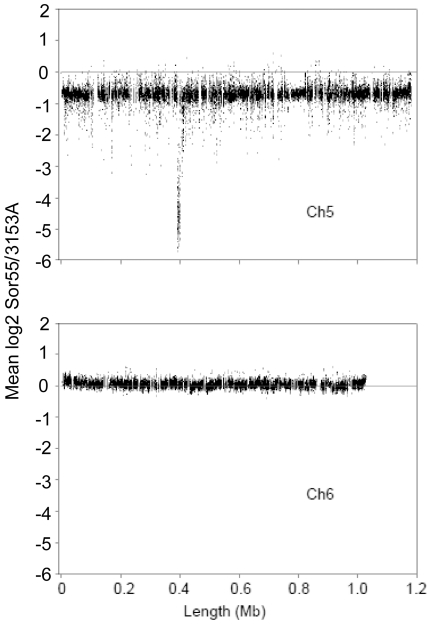
DNA profiling of the monosomic Ch5b by tiling aCGH. The mean aCGH log_2_ of the ratio Sor55/3153A for each of approximately 20,000 tiling probes on Ch5 is plotted according to the chromosomal position of the probe. Every point of the plot, thus, corresponds to a probe. Also presented is the similar plot of log_2_ of ratios for the Ch6 tiling probes. The horizontal line at position 0 on the ordinate corresponds to no DNA change. Note the global diminution of the DNA on Ch5b, but not on the control disomic Ch6 in the mutant Sor55. See Fig. 1 for the monosomic or disomic condition of, respectively, Ch5 or Ch6 in Sor55. Note that the absence of *MTL* ***a*** locus on Ch5b corresponds to dramatic diminutions on aCGH (see section “Limited analysis of gene expressions on another Ch5a, when it becomes monosomic in the strain Sor60” in Results).

It is important to note that there was no change in the size of the monosomic Ch5b, as determined with PFGE (Fig. 1 and “Evidence that the mutant Sor55 is monosomic for Ch5b”), even, though a large number of at least 186 genes were up regulated to the disomic level. If duplication of this number of genes occurred and duplicated copies were retained on Ch5b, considering the average gene length as 1 kb, Ch5b would become 186 kb longer. This value constitutes twice the difference between Ch5a and Ch5b in the parental strain 3153A, Fig. 1. Such a dramatic event is easily observed with PFGE. However, we have not excluded the unlikely possibility that the duplicated genes were transferred to various other chromosomes. Also, a small number of genes with the expression levels exceeding the disomic level, especially *PGA37* or *CAG1* that are up regulated, respectively, twofold or four fold above the disomic level, Table 2, raised the question of possible increase of the gene copy number of a subset of genes. Duplicated copies of the genes could reside on Ch5b or could be dispersed over different chromosomes. We, thus, investigated in more detail the copy number of the Ch5b genes in the mutant Sor55 by the aCGH approach with custom tiling arrays from Roche NimbleGen Inc. (Materials and Methods). The DNA content of Ch5b was estimated from approximately 20,000 Ch5 probes. When we calculated the ratio of averaged intensities Sor55/3153A for each probe from three arrays, we found, as expected, approximately a half global diminution of DNA across Ch5b. In Fig. 4, this result is presented by plotting averaged log_2_ of ratios Sor55/3153A for each probe according to the position on Ch5. Overall, slightly less than the expected half DNA diminution on Ch5b is explained by previously well-documented phenomenon of distortion due to dynamic range compression on aCGH [10]. By the contrast, an overall amount of DNA of the control Ch6 corresponded to the expected disomic level (see Fig. 1). Clearly, the Ch5b DNA content became monosomic with no large duplicated portions of the chromosome.

**Figure 5:**
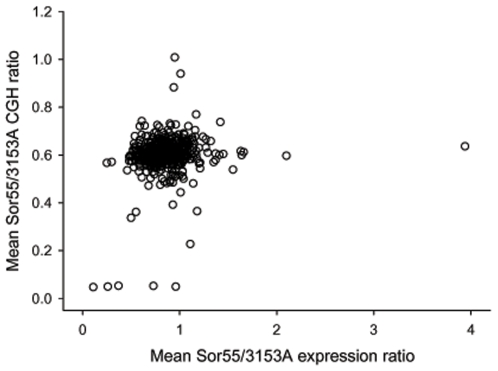
Combined plots for the expression and CGH ratios Sor55/3153A of Ch5b genes. The mean ratios Sor55/3153A for each Ch5b gene from the expression and CGH arrays are plotted, respectively, on the abscissa and on the ordinate. Every point of the plot, thus, corresponds to a gene. On the ordinate, three outstanding points that are positioned close to 1 indicate duplication of the genes orf19.2657, *CTA24* (orf19.4054), and *IMP2* (orf19.1981). The other five outstanding points that are positioned close to 0 correspond to the genes of MTL**a** locus that were lost on Ch5a in Sor55. The few points below 0.4 are indicative of the poor hybridization on aCGH. On the abscissa, most importantly, the larger majority of the points representing the genes, which are up regulated above the disomic level, expression ratio 1, are represented by a single copy, *i. e*., aCGH ratios ranging between 0.4 and 0.6. Also, all the remaining categories of the gene expression, down regulated, normalized, and intermediate are represented by the aCGH ratios that are tightly distributed around 0.5–0.6 for a single DNA copy.

Also, we combined probes mapping inside of ORF of each Ch5 gene and calculated the mean ratio Sor55/3153A for each Ch5b gene from three arrays (Table S2, Materials and Methods). From these data, we found at most only three genes on Ch5b, orf19.2657, *CTA24* (orf19.4054), and *IMP2* (orf19.1981), which possessed DNA/DNA ratios approximately 1.0 and which, thus, could be considered with certainty, as duplicated. Expression ratios Sor55/3153A of these genes, consistently, corresponded to the disomic level, Tables 2 and S2. As presented in Fig. 5, in which expression ratio of every gene is plotted against the gene DNA ratio, the points for these three genes are positioned on the ordinate clearly apart from the other gene values. A single gene, *SMD3* (orf19.4146), possessed DNA/DNA ratio slightly below 0.8 and was also expressed slightly above the disomic level, 1.17. Although not as clear, *SMD3* could be also considered as duplicated. Strikingly, the majority of the genes with high expressions exceeding the disomic level clearly possessed a single DNA copy; aCGH ratios raging between 0.5 and 0.6.

The large majority of genes, their expressions distributing between haploid and diploid levels, possessed DNA/DNA ratios distributed around 0.5 and 0.6, which is a clear indication of a single gene copy. A total of 62 genes possessed DNA/DNA ratios slightly varying around 0.7. However, we cannot interpret this ratio, as a clear indication of gene duplication, because the expression levels of those genes distributed over different levels including six genes that were down regulated to 50%. Taking into account the above, as well as the phenomenon of the compression on aCGH, we believe that most of those genes are not duplicated.

We confirmed the haploid copy number of four genes with Southern blot analysis. These genes were expressed at the monosomic or disomic level, *SEC14* or *MDJ1*, respectively, as well as twofold or four fold above the disomic level, *PGA37* or *CAG1*, respectively, Table 2. Each of these genes was used to prepare probes that were subsequently hybridized with blots containing either total digested DNA or native chromosomes (for detail see SI).

Our aCGH chips lacked a total of six genes that were, however, present on expression arrays, Table S2. Even if those genes were all duplicated, an unlikely possibility, a duplication of a minimum of only 10 genes or, if combined with some possibly duplicated genes with ratios 0.7 (see above), a maximum of, for example, 20 genes out of approximately 186 fully normalized genes is certainly not important for considering the generalization of widespread up regulation due to dosage compensation.

### Limited analysis of gene expressions on another Ch5a, when it becomes monosomic in the strain Sor60

We have asked whether dosage compensation also occurs on the alternative monosomic Ch5a. Similarly to Ch5b of the mutant Sor55, we confirmed the monosomic condition of Ch5a of the mutant Sor60 with electro-karyotyping followed by densitometry (Fig. 1; see above). The electro-karyotype was prepared with an aliquot of cells that were grown for RNA extraction. Semi-quantitative RT-PCR was used to prepare expression ratios Sor60/3153A from Ch5a with three genes representing three major expression levels on Ch5b. As presented in Table 2, we confirmed that, similarly to the Ch5b, on alternative Ch5a, *CAG1* is excessively over expressed, up to 5 fold, *HIS1* is normalized to the disomic level, and *PUT1* is approximately half-diminished.

## Discussion

Aneuploidy is responsible for many human genetic diseases, appears in most cancer cells and correlates with tumorigenicity. Clearly, in simple organisms, such as *C. albicans*, studies of gene expression in aneuploid cells, in particular of genes residing on aneuploid chromosomes, may facilitate the understanding of the mechanisms operating in higher eukaryotes and are of wide interest. Studying the expression of genes on monosomic Ch5 in *C. albicans* is also of special interest, as Ch5 monosomy is a means of survival under certain condition (see Introduction).

Statistical analysis of the genome-wide transcriptome responses to Ch5b monosomy revealed that 125 of 5,542 non-Ch5 genes (2%) significantly changed their expression level with p-values less than 0.01. These changes, however, did not lead to a decrease of overall chromosome expression. In contrast to these chromosomes, the monosomic Ch5b exhibited a significant decrease of expression to approximately 83–85% of the level of two chromosomes, but not 50%, as might be expected. The lesser decrease resulted from various changes in expression for different genes: decrease for some genes and no change or even increase for some other genes. Specifically, 15% of genes were down regulated approximately twofold (0.2–0.6); 40% were expressed at various levels between a twofold decrease and no change (0.7–0.8); 40% were fully compensated to the level of the disomic strain (0.9–1.1) (among these genes a few were duplicated, see Results); and 6% were up regulated above the disomic level up to twofold (1.2–2.1) with one peculiar gene, *CAG1*, up regulated four-fold (3.9). Thus, multiple regulatory mechanisms, not only Ch5b monosomy, are acting upon Ch5b genes in different ways. Multiple mechanisms are also implied by the normal distribution of the expression ratios Sor55/3153A of Ch5b genes shown in Fig. 1. The generality of dosage compensation on Ch5 was confirmed with a limited number of genes on the alternative Ch5a.

We assume that, as estimated by a rigorous approach, a large number of fully compensated transcripts on monosomic Ch5, 40%, is important for maintaining cellular homeostasis. Because these transcripts represent a large portion of the Ch5 genes, which are scattered along Ch5, the regulatory system responsible for this dosage compensation can be considered to be robust, performing global regulation across the chromosome. (In this regard, see below for dosage compensation in Drosophila).

An equally large group, 40%, of transcripts whose amounts range between monosomic and disomic levels, could result from different events: i) partial compensation in order to match to the expression of the other slightly down regulated genes of the corresponding pathways; ii) failure to fully compensate due to an incomplete execution of compensatory mechanism(s). The group of genes that are expressed between monosomy and disomy needs further clarification.

A relatively small group of genes, 15%, that are down regulated twofold or more contrasts with the approximately three times larger group of fully compensated genes. According to the Candida Genome Database (CGD) (http://www.candidagenome.org), many of the twofold down regulated genes are regulatory or metabolic and are implicated in either biosynthetic processes or translation, which is indicative of the overall decrease of metabolism of Sor55. Establishing more direct relevance of these genes to survival on sorbose medium needs further investigation.

While the twofold decrease could be explained by simple loss of DNA and represents a direct result of the monosomy, twofold and more up regulation of genes from another outstanding small group, 6%, could be due to interactions with other genes on various chromosomes representing an inverse affect of monosomy. A specific nature of these up regulated genes may be related to adaptation to sorbose; however this relationship at present remains obscure.

Surprisingly, there is a striking similarity between our data and some of the results with cancer cells. Platzer *et al*. [11] analyzed four major amplifications of entire chromosomes or chromosome arms 7p, 8q, 13q, and 20q in liver metastases of colon cancer. Among more than 2,000 unique array targets within amplified chromosomes, only a small fraction, 3.8% of the genes, was up regulated twofold, two genes were up regulated five-fold, and, surprisingly, 7.7% of the genes were down regulated twofold, whereas the vast majority of the genes were expressed between twofold decrease and twofold increase suggesting that they are partially or fully compensated to the disomic level, despite the chromosomal amplifications. In the prostate tumor cell line, Phillips *et al.* [12] identified multiple chromosomal rearrangements including duplications, amplifications, loss of entire chromosomes or chromosome arms, as well as non-reciprocal translocations. An overall significant association of approximately one half of down or up regulated genes with, respectively, DNA gain or loss was established. Although, changes in expression of the genes mapping within the aneuploid regions ranged from less than twofold to more than twofold, as compared to the disomic level, only a few genes were up regulated more that twofold on duplicated or amplified chromosomes. Also, there were cases of inversely regulated genes, 14% down regulated in regions of DNA gain and 9% up regulated in regions of DNA loss.

Similarly, Makarevitch *et al*. [13] recently found selective dosage compensation on a chromosome acquiring triploid arm in plants: 40% of the genes were up regulared at a level that is expected for the trisomy, 1.5-fold; however, 60% exibited no changes, while a few genes were up regulated more than twofold compared to the disomic level.

Dosage compensation has been predominantly studied with heteromorphic sex chromosomes in species in which females possess two gene-rich large X chromosomes (XX), whereas males possess one X and one gene-poor small Y. Various mechanisms equalizing X genes with genes on autosomes and between males and females were uncovered in mammals, the fly *Drosophila melanogaster* and the worm *Caenorhabditis elegans*. For example, one X in female mammals is practically silent; while the active X is globally up regulated in both females and males. The latter is in contrast to the fly or the worm where the single X in males is up regulated, whereas in the XX hermaphrodite worm, both Xs are partially repressed [14]–[16]. Dosage compensation for sex-chromosomes is thought to have evolved in order to prevent debilitating gene disbalance, as dramatically exemplified with various human diseases due to aneuploidy of sex-chromosomes, autosomes or portions of chromosomes [17], [18]. Surprisingly, recent studies with several species of birds having ZZ males and ZW females have challenged this idea [19], [20]. The dosage compensated and non-compensated genes occurred across Z in females, diminishing the overall Z expression to approximately 80% of the ZZ level in males.

Dosage compensation in the fly is currently well understood. The males-specific lethal (MSL) complex of proteins and non-coding RNAs binds to hundreds of sites along X in transcription-coupled fashion allowing a key component of MSL, a protein MOF, to acetylate histone H4 at lysine 16. The activity of MSL is thought to facilitate hyper-transcription by leading to more open chromatin structure. Furthermore, transcription-coupled methylation of histone H3 at lysine 36 enhances recruitment of MSL [21], [22]. On the other hand, in females dosage compensation is inhibited by the female-specific RNA-binding protein Sex lethal (SXL) [23].

In contrast to higher eukaryotes, dosage compensation at the transcriptional level has not been found in lower eukaryotes, such as the yeast *Saccharomyces cerevisiae*. By studying large collection of laboratory strains, Hughes *et al.* [24] identified twenty aneuploid strains, in which expression of nearly every gene on a trisomic or a monosomic chromosome was correspondingly altered. Torres *et al*. [5] created a collection of haploid *S. cerevisiae* strains that each bear an extra copy of one or more of chromosomes. An approximate doubling of gene expression along the entire length of duplicated disomic chromosomes was observed. Although no compensatory mechanisms at the level of transcription was revealed, the authors found that the amounts of many, but not all analyzed proteins, did not increase, thus, indicating the cell's attempt to restore its normal physiological state.

In this study, we demonstrated that in a lower eukaryote *C. albicans*, regulation by Ch5 monosomy is coupled with a widespread compensation of the gene dosage at a transcriptional level. Such coupling obviously diminishes detrimental consequences of aneuploidy, while allowing a decrease or increase of specific transcripts.

## Materials and Methods

### Strains

The laboratory strain 3153A has been largely used to study chromosome aneuploidy [1]. Its derivative Sor55 is a representative mutant that is monosomic for Ch5b [1], [6], whereas the derivative Sor60 is monosomic for Ch5a.

### Custom designed expression microarrays from CombiMatrix Corporation

CombiMatrix Corporation (Mukilteo, WA) designed DNA microarrays, as based on 6,321 open reading frames (ORF) found in assembly 19 of the *C. albicans* genome sequence (http://sequence-www.stanford.edu; http://www.candidagenome.org/). The “CustomArray™ 12 K” microarrays have been provided to us by CombiMatrix Corporation (http://www.combimatrix.com/products_customarray.htm). The CustomArray™ uses a specially modified semiconductor where electrical currents delivered to the platinum pads create a localized proton flux that acts as a chemical deprotectant for in situ phosphoramidite synthesis. The reactions on each pad are digitally controlled and allow directing the synthesis of oligonucleotides. The lengths of 90% of the oligonucleotides are between 35–40 nucleotides; whereas the other 10% are longer. The oligonucleotides were predominantly designed to correspond to the 5′-and 3′-regions of the ORFs. Furthermore, out of the approximately 80 single nucleotide polymorphisms, which map to Ch5 sequences, only one occurred in the same location as one of the probes. Each chip contains 11,898 unique probes that are spread across 12,000 spots. The ORFs on Ch5 are represented at least twice. Each chip also contains 544 standard control probes, of which 395 are exogenous sequences not found in *C. albicans*.

### Hybridization to expression microarray chips and handling of chips

Three batches of total RNA were prepared for each strain, using independent cultures (see Supporting Information S1 for more detail). 2 µg of total RNA from each batch was used to synthesize Cy5-dUTP labeled anti-sense RNA, using the Ambion Amino Allyl Message Amp II aRNA Amplification Kit (Ambion, Austin, TX). Anti-sense RNA was hybridized to an individual chip as recommended by CombiMatrix Corporation (http://www.combimatrix.com); the hybridization was carried out in the Functional Genomics Center at the University of Rochester Medical Center. The chips were scanned with a ScanArray Lite microarray laser scanner (Perkin Elmer, Boston, MA) at a 5 µm resolution. The resulting 16-bit TIFF files were quantified with Microarray Imager software (CombiMatrix Corporation, Mukilteo, WA).

### Analysis of transcriptional profiling by DNA microarrays

Data from three independent experiments were combined and statistically analyzed with a custom-designed program. Statistical analysis scripts were written in R for normalization, ANOVA, significance tests, and visualization. Genome annotation was parsed from CGD. Because each ORF from Ch5 was spotted twice on each chip, six signal intensity ratios Sor55/3153A were compared. The signal intensities of the 395 control non-*C. albicans* probes (see above) were treated as non-specific hybridization and the median of their values on each chip is taken as the background signal. Because the standard deviation of negative control probes is approximately 200 on all chips, genes with expression levels <200 are considered in the same levels as background noise and are excluded from estimation of expression level changes. The signal intensities of annotated genes are obtained after subtraction of the background signal from their raw values. The background-corrected signals are then normalized by assuming that the total combined signals of non-Ch5 genes on each chip remain constant, because genes on monosomic Ch5 are expected to change their expression levels. Specifically, the scaled signal for each gene is:
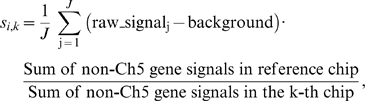
where *s_i,k_* is the signal for gene i on k-th chip, and *J* is the number of probes for gene *i*. This constant normalization method (or scaling method) is often the most biologically consistent method [25]. When calculating the expression ratio Sor55/3153A, negative signals are considered as 1. The final ratio is the average of the paired hybridizations experiments. Pair-wise t-test was performed using the base-2-log transformations of the scaled-signals from three hybridizations in both Sor55 and 3153A. Raw data are available at Gene Expression Omnibus (GEO), http://www.ncbi.nlm.nih.gov/geo/, with the accession numbers GSM455127-GSM455132.

### Custom designed CGH microarrays from Roche NimbleGen Inc

Roche NimbleGen Inc. (Madison, WI) designed tiling DNA microarrays for the *C. albicans* genome supercontigs (assembly 19 from the CGD). The HX3 microarrays have been provided to us by Roche NimbleGen Inc. (http://www.nimblegen.com/). Each microarray contains 710,907 probes that were permitted to match up to four times within the genome. Of all probes, approximately 20,000 matched the Ch5 sequences. The probes were *in-situ* synthesized 50-mers with an average probe spacing of 35 bp on each strand. The design was tiled in an unbiased fashion across the entire genome. The number of probes per gene varied depending on the size of the gene. Empty features on the array were filled with randomly generated probes that had G+C content and length comparable to the *C. albicans* probes and that acted as controls for non-specific binding.

### Hybridization to CGH microarrays and handling of chips

In order to increase statistical confidence, independent batches of total DNA were prepared using independent cultures for each strain, Sor55 or 3153A. A total of 10 µg of DNA from each strain were sonicated per Nimblegen's instructions, using a Branson model 450 Sonifier, resulting in a mean size of ∼500 bp. The sheered DNA was quality checked on a 2% agarose gel. Labeling was performed as recommended by Nimblegen, using, as two separate 2 µg of DNA reactions for each strain with either Cy3 or Cy5 conjugated random nanomers for 2 hours at 37°C. The labeled targets were dried and re-suspended in NimbleGen hybridization buffer. A total of 31 µg of each labeled DNA samples, Sor55 and 3153A, were combined and hybridized with each array at 42°C for 72 hours in the NimbleGen Hybridization System. The arrays were washed, as recommended by manufacturer, and dried using an Array-It mini-centrifuge. The arrays were scanned on a NimbleGen MS 200 Microarray Scanner at a resolution of 2 µm (49 pixels/13 µm feature) and resulted in a 40% increase in signal/noise, as measured by mad.1dr. Signal intensities were extracted from the scanned images using NimbleScan 2.5. Briefly, spatial correction was applied to adjust for possible signal intensity variance across the surface of the array then normalization was performed to compensate for differential signal intensities between Cy3 and Cy5 dyes.

### Analyses of genome profiling by aCGH microarrays

Functional Genomics Center at the University of Rochester Medical Center analyzed hybridization data from two independent DNA batches of 3153A or Sor55 that were labeled, respectively, with Cy3 or Cy5, as well as one dye-swap. The normalized intensity ratios Sor55/3153A for each probe were averaged from three arrays to form a single data point. Data were highly consistent between three arrays. The Sor55/3153A ratios from these genomic probes reflect the relative copy number of the corresponding genomic loci. In addition, the mean ratio Sor55/3153A for each gene on Ch5b was prepared from the mean values of the probes tiling inside of the corresponding ORF. The number of such probes per ORF varied dependent on the gene size. Raw data are available at GEO with the accession number GSE21616.

### Northern blot analysis

General approaches for Northern analysis were adopted from Ding *et al*. [26]. Each transcript was examined at least 3 times using different RNA batches and different internal controls. Images were visualized using PhosphorImager Storm-820 (Molecular Dynamics, Sunnyvale, CA). Individual bands were quantified using ImageQuant 5 software (Molecular Dynamics, Sunnyvale, CA). For more detail see SI.

### RT-PCR analysis

Total RNA was additionally treated with RNase free DNase to remove all traces of genomic DNA. Semi-quantitative RT-PCR analysis is described in [27], [28]. Briefly, each transcript was examined from several different RNA batches and same or different internal controls. Control genes were selected according to the following criteria: residing outside Ch5; no expression change in Sor55 and similar expression level to the studied gene; the absence of amplification inhibition and non-specific bands while co-amplifying; a close match of the number of cycles required for both amplicons to reach the exponential phase [28] (see SI for the enzyme use and for more detail). Genes of interest and control genes were amplified in the same tube for different number of cycles, Fig. S1. The images of amplicons from several consecutive cycles in exponential phase were used for densitometry. Studied gene was normalized against the control gene by calculating mean ratio of densitometry values.

### Various procedures

For preparation of total RNA see SI. Preparation and analysis of electro-karyotype, cell transformation, as well as procedures that diminish undesirable chromosome instability were carried out, as previously reported [3], [29]. For genes and primers used in this work see Table S1.

## Supporting Information

Supporting Information S1 (0.03 MB DOC)

Figure S1 Analysis of RT-PCR products amplified from total RNA of strains 3153A and Sor55 using primers for CAG1, orf19.4349, and URA4.
(1.12 MB TIF)

Table S1 List of genes and primers.
(0.07 MB DOC)

Table S2 Expression arrays^a^ and aCGH values calculated as mean ratios Sor55/3153A for all Ch5b genes.
(1.05 MB DOC)

Table S3 RT-PCR validation^a^ of the expression array values with 30 genes^b^ on Ch5b in Sor55.
(0.05 MB DOC)

Table S4 Northern blot validation of the array expression values with 18 genes^a^ on Ch5b in Sor55.
(0.03 MB DOC)
