# Supplementary material for: Widespread Occurrence of Dosage Compensation in Candida albicans
Source: PLoS One. 2010 Jun 11;5(6):e10856. doi: 10.1371/journal.pone.0010856 (PMC2883996; doi:10.1371/journal.pone.0010856)
Supplement: Supporting Information S1 — (0.03 MB DOC) [file pone.0010856.s001.doc]

# Supporting Information

**Total RNA extraction.** 3153A or Sor55 cells were removed from –70° C stocks and grown at 37° C as independent colonies, approximately 3000 colonies per plate, on synthetic medium containing yeast extract, peptone, and 2% sorbitol instead of glucose (for detail, see synthetic dextrose medium in 1). 2% (wt/vol) agar was also added. Colonies were harvested, washed, and RNA was isolated as previously described (2). RNEasy clean-up columns (Qiagen Inc., Valencia, CA) were used to remove the 5S RNA.

**Determination of gene dose for four representative genes on monosomic Ch5b using Norther blot analysis**. In order to determine the genes copy number, we employed two kinds of Southern blot analyses. First, we immobilized the cells of the parental strain 3153A and its derivative Sor55 monosomic for Ch5b in agarose plugs and treated the cells according to a standard procedure for preparation of intact chromosomes (3). A unique restriction site, either HindIII or KpnI, was identified within ORF of each the following gene: *CAG1* (orf19.4015), *PGA37* (orf19.3923) or *MDJ1* (orf19.6672) and corresponding enzymes were used to treat the agarose plugs according to manufacturer’s instructions. Digested DNA was separated with conventional electrophoresis, blotted, and Southern analyzed with corresponding probes, which were prepared from the above genes using ^32^P-labelling with NEBlot™ kit (New England Biolabs, Ipwich, MA). Each probe generated two signals, as expected if restriction fragments occur between a site within ORF and a chromosomal site on each flank. The sizes of two fragments were as expected. Signal patterns of 3153A and Sor55 were identical. Moreover, signals from Sor55 always were approximately half as bright as from 3153A. These results indicate no tandem gene duplication on monosomic Ch5 or generation of a second copy with the following transfer to another chromosome, as well as they are indicative of no duplication of a larger portion on monosomic Ch5. Alternatively, we separated chromosomes of 3153A and Sor55 side by side in PFGE under running conditions optimizing separation of Ch5 (4, 5); blotted several gels, and hybridized the blots with the probes prepared from *CAG1*, *PGA37*, and *SEC14* (orf19.941). While signals came only from Ch5, the brightness of signal from monosomic Ch5b of Sor55 was consistent with such from either Ch5a or Ch5b of 3153A. Although, an extra signal with *PGA37* came from one of the long chromosomes indicating an extra-copy, the signal and its brightness was the same in both Sor55 and 3153A. [We have previously reported an extra-copy of *LYS1*, which was carried on one of the long chromosomes in another *C. albicans* laboratory strain (6)]. These results are consistent with no increase of the amount of DNA for the analyzed genes.

**Validation of expression arrays with Northern blot analysis.** A total of three Northern blots were prepared for each gene at a separate time using a separate batch of RNA. Briefly, approximately 10 μg of total RNA were size fractioned on a 1% denaturing gel, and then transferred to positively charged nylon membrane (Ambion, Austin, TX). Blots were hybridized with probes in QuikHyb Hybridization solution (Stratagene, LaJolla, CA) overnight at 42° C. The probes were PCR amplified from genomic DNA of the strain 3153A with corresponding primers (Table S1) and were radiolabeled using NEBlot kit (New England BioLabs, Ipswich, MA). 18S RNA, *PGK1* (orf19.3651), and *VPH1* (orf19.6863) were used as loading controls. Before the next probing, the membranes were stripped twice for 15 min in 0.2% SSC and 0.1% SDS at 80° C. Stripping was confirmed by re-exposure, and membranes were hybridized again with different probes, but not more than four times.

**Validation of expression arrays with RT-PCR analysis.** RT was performed with oligo-dT_21_ primers using MonsterScript Reverse Transcriptase (Epicentre Biotechnologies, Madison, WI) as recommended by manufacturer. All PCRs were done in 50-μl volumes with Phusion Hot Start High-Fidelity DNA Polymerase (Finnenzymes, distributed by NewEngland BioLabs, Ipswich, MA) in accordance with supplier’s protocol. The following genes were used as controls: *PDI1* (orf19.5130); *VPH1* (orf19.6863); *SPE3* (orf19.2250); *GAD1* (orf19.1153); *SUP45* (orf19.3541); *SUP35* (orf19.1378); *RPL16A* (orf19.6085); *SSC1* (orf19.1896); *QCR8* (orf19.4490.2); orf19.5063; *TPI1* (orf19.6745); *ACT1* (orf19.5007); *CDC6* (orf19.5242), and orf19.1190. The PCR products were analyzed on a 3.0% agarose gels stained with ethidium bromide. The images of amplicons were captured by AlphaImager IS2000 Digital Imaging System (Alpha Innotech Corp., San Leandro, CA) and quantified using ImageQuant 5 software (Molecular Dynamics, Inc., Sunnyvale, CA).

# References

1. Sherman F (2002) Getting started with yeast. *Meth Enzymol* 350:3-41.

2. Russo P, Li WZ, Hampsey DM, Zaret KS, Sherman F (1991) Distinct *cis*-acting signals enhance 3´-endpoint formation of *CYC1* mRNA in the yeast *Saccharomyces cerevisiae*. *EMBO J* 10:563-571.

3. Ahmad A *et al.* (2008) Chromosome instability and unusual features of some widely used strains of Candida albicans. *Yeast* 25:433-448.

4. Kabir MA, Rustchenko E (2005) Determinantion of gaps by contig alignment with telomere-mediated chromosomal fragmentation in *Candida albicans*. *Gene* 345:279-287.

5. Wellington M, Rustchenko E (2005) 5-Fluoro-orotic acid induces chromosome alterations in *Candida albicans*. *Yeast* 22:57-70.

6. Perepnikhatka V *et al*. (1999) Specific chromosome alterations in fluconazole-resistant mutants of *Candida albicans*. *J Bacteriol* 181:4041-4049.
