# Supplementary figures and images for: Widespread Occurrence of Dosage Compensation in Candida albicans
Source: PLoS One. 2010 Jun 11;5(6):e10856. doi: 10.1371/journal.pone.0010856 (PMC2883996; doi:10.1371/journal.pone.0010856)

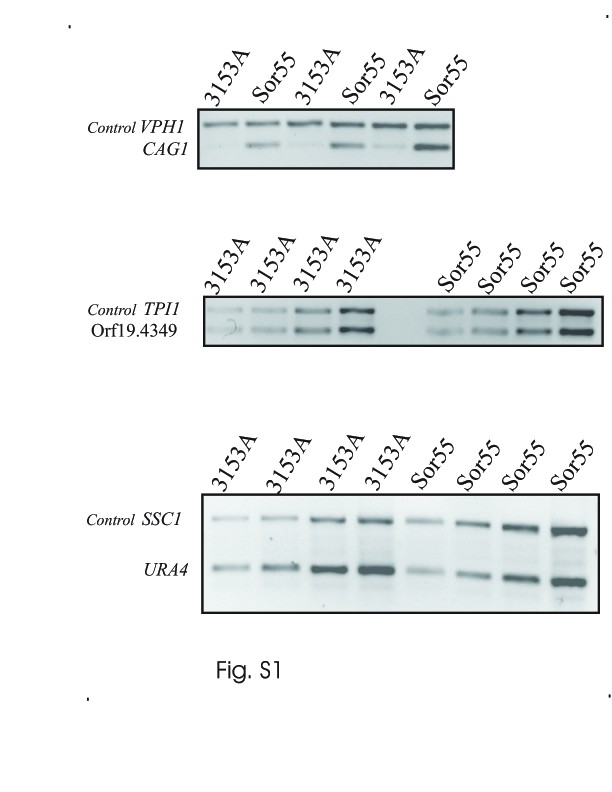

Supplement: Figure S1 — Analysis of RT-PCR products amplified from total RNA of strains 3153A and Sor55 using primers for CAG1, orf19.4349, and URA4. (1.12 MB TIF) [file pone.0010856.s002.tif]
