# Supplementary material for: Widespread Occurrence of Dosage Compensation in Candida albicans
Source: PLoS One. 2010 Jun 11;5(6):e10856. doi: 10.1371/journal.pone.0010856 (PMC2883996; doi:10.1371/journal.pone.0010856)
Supplement: Table S1 — List of genes and primers. (0.07 MB DOC) [file pone.0010856.s003.doc]

# **Table S1.** List of genes and primers

| Gene | Primer |
| --- | --- |
| *EMP24*  (orf19.6293) | N-EMP24F1; 5’-GGGTTGGACCATGGTGACGAAA  N-EMP24R1; 5’-TTGTTCGGCTCTGACATCATCG |
| orf19.2087 | S-F; 5'-GTCACATTTGGTAAATACGAGTTTCC AS-R; 5'-CGACTCCGGGAACAACCTTG |
| 18S rRNA | S-F: 5'-TTCTGCCCTATCAACTTTCGATGG AS-R: 5'-CAGACCTCTCGGCCAAGG |
| *PGK1* (orf19.11135) | COM97F; 5’-CTTCCAAAGACAAGGATGGTAAGAAAGTCAAG COM98R; 5’-ATTTCTTAGCAACAGTAGCAGTATCACCACCA  *140PGK1F1; 5’-TTGGATGCTGCTGTCAAATCTGCTG  *141PGK1R1; 5’-AATTCCAATGAAGCACCACCACCA |
| *VPH1* (orf19.6863) | COM115F; 5’**-**GTGAAACCACCAACGATAATGAAGAAACAG  COM116R; 5’**-**CAGCTGCTAAAGTAACAATGAAACCATGACC  *VPH1-Q12F; 5’-TGCATTTGGTCCCACCGGTTTA  *VPH1-Q12R; 5’-AAAGCAGAACCACCACCTTCAAA |
| *SPE3*  (orf19.2250) | *Hg-SPE3F1; 5’-CCGATACCATGTGGCCAGGTCAA  *Hg-SPE3R1; 5’-GCCAAGCTTCTTCAATTGATTCGTG |
| *GAD1* (orf19.1153) | *Hg-GAD1F1; 5’-ATTGGTACCGCAACCACCGGTTC  *Hg-GAD1F1; 5’-GCACCAGTGAAAGTTGATCCCATGA |
| *SUP45* (orf19.3541) | *Hg_SUP45F1; 5’-CGGTACCGCCTCGAACATTAAGTCA  *Hg_SUP45R1; 5’-TGACAGCACCAAACAATGCACCA |
| *SUP35* (orf19.1378) | *Hg-SUP35F1; 5’-TGGTGGTGCATCTCAAGCTGATG  *Hg-SUP35R1; 5’-TCAAACCAGCACCGGTGTAACCA  *Hg-SUP35F2; 5’-TGGCCAATGCATCTTTGAATGGA  *Hg-SUP35R2; 5’-CTGTTGCTGCTGTTGAGGCTGGA |
| *RPL16A* (orf19.6085) | *Hg-RPL16F1; 5’-GGCCATTTGTTGGGTCGTTTAGCC  Hg-RPL16R1; 5’-CGTATGGTGGTGGGACACCTTCG |
| *SSC1* (orf19.1896) | *SSC1F2; 5’-TGCTGGTTTATCCACCTCCGATG  SSC1R2; 5’-GGATGGCAGCACCCATAGCAACA |
| *QCR8* (orf19.4490.2) | *Hg-QCR8F1; 5’-TTCGTTACGCTGCCACTCCTGCT  *Hg-QCR8R1; 5’-GCTTCAGCATTAGCTTGAGCATTTC |
| orf19.5063 | *Hg-5063F1; 5’-TGCTGCCACTTCCGTTTCATCAT  *Hg-5063R1; 5’-TCCACATTCACTAGTTTCAGCTTCA |
| *TPI1* (orf19.6745) | *TPI1-Q9F; 5’-GCCAGACAATTGGATGCTGTTTCC  *TPI1-Q9R; 5’-GCTTGTTCGGCACCAATGCTCT |
| *ACT1* (orf19.5007) | S-F: 5'-GACAAATGGGTAGGGTGGGAS-R; 5'-CCAATAGTGATAACTTGACCATCTGG *ACT1-Q1F; 5’-GGTGACGACGCTCCAAGAGCTG  *ACT1-Q1R; 5’-TCGTCCCAGTTGGAAACAATACCG |
| orf19.1190 | Hg-1190F1; 5’-AGAAGAACAGGCCCTTGCGATGC  Hg-1190R1; 5’-TGACAATTGTGCGTGTGCCAAGG |
| *CDC6*  (orf19.5242) | *NSU-CDC6-F1; 5’-CCTTCAACTTCACCTTCAATTCCAA  *NSU-CDC6-R1; 5’-CAGTCTTACCCGTCCCTGGTGGA  *NSU-CDC6-F2; 5’-CACCAACCAAATCCAATAACTCACG  *NSU-CDC6-R2; 5’-CAGTCTTACCCGTCCCTGGTGGA |
| *RPS25B* (orf19.6663) | COM-63F 5’-GGTAAAGTTAAGGACAAGGCTCAACACATTG  COM-64R 5’-CGAGTGTAGATAGCTTGTTTGGAGTGTTTCA |
| *RIB5* (orf19.4024) | COM151F 5’-CTACTCAAAGCACGATGATTCCTCCACTGGT  COM152R 5’-ATGTTGACAGTGTCGCCTACTTCCTTCTTGG |
| *SQT1*  (orf19.4029) | COM153R 5’-AGGTGAACAGTGGGTGAAATTTGGTGAATTG  COM154R 5’-GACCAATGAAACACCTTCATCACCAGCAGTA |
| *ECM331* (orf19.4255) | COM147F 5’-ATAGTCGCTGCCTTATTGACATCAGTTTCAGCA  COM148R 5’-AGAAATGGTGACAGCACCTTTGATAGTGGTCAA |
| *ERG11* (orf19.922) | S-F: 5'-GGCTATTGTTGAAACTGTCATTGATGG AS-R: 5'-CAGCAGTATCCCATCTAGTTGGATC |
| *RPS11A*  (orf19.4149) | COM149F 5’-ggtgacaacatcaccttcttcaactctgaaagc  COM150R 5’-gtccattcgctggtactgtttccatcagaggta |
| *ADH1* (orf19.3997) | COM-67F 5’-GTTGATTCACGTCAAATACTCTGGTGTCTGT  COM-68F 5’-GCATTTGATTAAACCTCTGGAGAAGAAGTCA |
| *INT1* (orf19.4257) | INT1-F; 5’-GTTGAGCCACCCTCAATACAACATCAATGG  INT1-R; 5’-ACCTTCTTAGGTGACCCAGGCGTTGATTCT |
| *TEF4* (orf19.2651) | COM71F; 5’-TGATGGATTCCAATTAACTGAAACAATAGCC  COM72R; 5’- CAGGATAAGTCAATTGCTGTTGAGGAAACTTTA  *N-TEF4F; 5’-TTAGTGGCTATTTAGTTGGGGACAA *N-TEF4R; 5’-CAGGATAAGTCAATTGCTGTTGAGG |
| orf19.5686 | COM97F 5’-GTTCTATGAAGCTGATCAGAAGTTGGACCAT  COM98R 5’-CCATGGTTTAACCTAACCTTATCCCAAACAG  *N-19.5686-F; 5’-GTTGGACCATGCCGAGAGGGTTG  *N-19.5686-R; 5’-TTCCCAGCCAACAACGTCCCAAA |
| *CTA24* (orf19.4054) | COM81F 5’**-**CACTCGACGAGATATTGAAATCATCAGGATAC  COM82F 5’**-**CTGTCTACATCAAACTCTTCATTTACGTGGTC  *N-CTA24F1; 5’- gccgccccacaaaag tttgac  *N-CTA24F1; 5’-tcatttacgtggtcgccattgtc |
| orf19.4248 | COM85F 5’-CTCATAGTTGGTATTGTCTTCCAGCAACTTTC  COM86R 5’-CTGGCACTATGAGCCACACCTTCATAAGTATT  *N-19.4248-F; 5’-tggtggctgtgttgctttatcagga  *N-19.4248-R; 5’-CGGCCAATTCTTCTTCACTGGCACT |
| *HIS1* (orf19.4026) | S-F: 5’-GGATTTAGTCAATCATTTACCAGACCAs-R: 5’-GATATCTCGAGTACCAATATATCGGTTG *N-HIS1F1; 5’-cgacaacatcgaggacttgttgga  *N-HIS1R1; 5’-cagcaacacccaaggcacaaga |
| *PRE1* (orf19.4025) | COM89F 5’**-**ATTGCAACATCTAAAGCTGCCACAAGAG  COM90R 5’**-**GGCATTCTCGTTTGTAATTCCTTGACAC  *N-PRE1-F; 5’-ggtgatacggttcagtttgccgagt  *N-PRE1-F; 5’-atgcagcgtagccatgagcacca |
| *COR1* (orf19.4016) | COM129F 5’**-**GCCTCCATTGCTTCCAATGCTGTTGATATT  COM130R 5’**-**CCATCTCATCATGGCCATTTCATTTCTGTT  *CM-COR1-F 5’-GCCAGTGCAGTTGAAGCTGATCC  *CM-COR1-R 5’-GGCCAAGTGACGCAAGGAGTCTT |
| *PGA56/CSU51* (orf19.1105.2) | AF166-F; 5’-ATGGAGCATTGGTTAATGGACCGACTG  AF167-R; 5’-GTAAGACTTTCCTTGCTGGTGTTGGTGCTGG  *NSU-6312F1; 5’-TGCCGGTGCTAAAGGTGTTGAAGT  *NSU-6312R1; 5’-TGGACCAAATCTATTAGCAGCTGGA |
| *YNK1* (orf19.4311) | COM-69F; 5’-TCTTGGGTAGATTTGAACAAAGAGGTTTCAA  COM-70R; 5’-TTAGCAGATTCAACAGAATCAGAACCATGAC  *D-YNK1F1; 5’-ACCAGACGGTGTTCAAAGAGGTTT  *D-YNK1R1; 5’-CCTGGAGCAGATTGTAATGGGTTAG |
| *MDJ1* (orf19.6672) | *N-MDJ1F1; 5’-ctcatgtcatgggtgggttcca  *N-MDJ1R1; 5’-attggagcatcaccggcaccag  Au- MDJ1F; 5’-ACTTGGCCACAAGAGCATTTCACTCATC  Au- MDJ1R; 5’-GATGGACACGTGGAAGACATATGGAACC |
| *PUT1* (orf19.4274) | *D-PUT1F1; 5’-TGGCTTAAGATTGCATGAACGAGGT  *D-PUT1R1; 5’-TTGCTGCATCTTTAGCAAATCCTG  Au-PUT1F; 5’-ACTGGCTTAAGATTGCATGAACGAGGTA  Au-PUT1R; 5’-CACTGGCTAATCTCATTGATTCAGCAT |
| *SAH1*  (orf19.3911) | *CM-SAH1F; 5’-AAGTTGCCATTGTTGCCGGTTTC  *CM-SAH1R; 5’-TGGCATCTTCTGGCATTTGTTCG |
| *UBC13* (orf19.5337) | *N-UBC13F; 5’-TCAATCACCTTATGCCAAGGGGAAA  *N-UBC13R; 5’-GCCAAAGGATCATCTGGGTTTGG |
| Orf19.3216 | *D-3216F1; 5’-CGCAACAATCACAATTTCAAACACC  *D-3216R1; 5’-TTTGGTACCAGTTGTTGTTGCTGT |
| *SEC14* (orf19.941) | *D-SEC14F1; 5’-cctgcatgttcaagaaaagctggt  *D-SEC14R1; 5’-tgcagtagaaaacccaaatggagca  Au- SEC14F; 5’-TCCAACCGATCAAACAGGTTACACATCA  Au- SEC14R; 5’-CGTCATCAGAAACATCTGACATACCACCA |
| *THS1*  (orf19.5685) | *D-THS1F1; 5’-TTTCCAACAGGACGATGCCCATA  *D-THS1R1; 5’-CCCATTTGCCTTCACCCAAGAAA  Au-THS1F; 5’-AGCAAGCCTTAAACGATTTCTTGGGTGA  Au-THS1R; 5’-CTTCTCTTGGATTCTCTCAACGCAACCA |
| *VIP1* (orf19.3895) | *D-VIP1F1; 5’-GACCGACCTGCGCTTGTCAATTA  *D-VIP1R1; 5’-TGAGTGTGACAACCTGGCGACAA |
| *GDS1* (orf19.1963) | *D-GDS1F1; 5’-cgtggatctactccgcctacatcg  *D-GDS1R1; 5’-gcaattggtgctcgagaaattgg  Au-GDS1F; 5’-GCTCCTGGTTGGGAAGTCAAATTGAAAG  Au-GDS1R; 5’-CACCAGTTTCTCCTAATGGAGCCCAAGT |
| *URA4* (orf19.1977) | *D-URA4F1; 5’-TGATGCCGTTGAAACTGTTCGTGA  *D-URA4R1; 5’-GCATGAATCGGATGTGGCGCACT |
| orf19.4349 | *N-KRE2F; 5’-TCACCAAGGCAAGAACAATCTCCAA  *N-KRE2R; 5’-ACCCATCATCGTCATCGTCGTCA |
| *ACH1* (orf19.3171) | *N-ACH1F; 5’-GGGCCAGTGCTGGACCTGAAGAA  *N-ACH1R; 5’-CCAACAGCTGGACCAGGAACAATGG |
| *RPO26* (orf19.2643) | *N-RPO26F; 5’-GCTCAAGCAGCTGGTCAACCAAGAA  *N-RPO26R; 5’-CCCAATCTTCGTAAGATCCATCAGG |
| *GLR1* (orf19.4147) | *N-GLR1F; 5’-TGGAGATTTCGATTGGGCTAAACTT  *N-GLR1R; 5’-GCTGTACCACCAGTGGCAATCAA  Au- GLR1F; 5’-TACATCTGATGGGTTCTTTGCCTTGGAA  Au- GLR1F; 5’-CCAATAGAACCAGCTTCTGGATGTGAGA |
| orf19.4220 | *CM-H4220-F 5’-cacgtttgttcgtcagagggatga  *CM-H4220-R 5’-tcattcctgttgaaattcgcaagc |
| *PGA37* (orf19.3923) | *UP-PGA37F; 5’-CAAGTGGCAGCTCGAGCAGTGG  *UP-PGA37R; 5’-ATCCACAAGTGCTTCCTGAGCAATG |
| *GAP1* (orf19.4304) | *CM-H4304F; 5’-caacatcaatgctgccaattcca  *CM-H4304R; 5’-tccaccagaaactgggaatgcaa |
| *CAR1* (orf19.3934) | *CM-H3934-F; 5’-GCTGTCAAGGGAAGTTTGGCAGA  *CM-H3934-R; 5’-TGGACAACCGTGTAAATTGCCTGA |
| *GAP6* (orf19.6659) | *CM-H6659F; 5’-AAACCAGGCATGTGGCGAAACTT  *CM-H6659R; 5’-CCGGACGCCACTAATAACCCTGA |
| *CAG1* (orf19.4015) | *UP-CAG1F1; 5’-ggtgggtttacccaacaggagaga  *UP-CAG1R1; 5’-tctgtaccaccagcaacatcagca |
| *SUI1* (orf19.1280) | *N-SUI1F1; 5’-TTCGCCGACACTGGTGATTCAG  *N-SUI1R1; 5’-TTGGATAACTTCACCCAATTCATCA |
| *SCW1 (* orf19.3893) | *D-SCW11F; 5’-ttggatatctgctgctggtgttga  *D-SCW11R**;** 5’-tggcaggtggttcagaagtggtga |
| *MTLALPHA1* (orf19.10712) | MT-ALPHA1-F1; 5’-CGACGAGCCGTATTCCCAAGAGC  MT-ALPHA1-R1; 5’-TGTTGCAGGCGAAGACTGGAGTTG  AF140-F; 5’-CCAAGAACTATTTCCAATCCGGAA  AF141-R; 5’-AACATCCTCAATTGTACCCG |
| *MTLA1* (orf19.3201) | MT-A1-F1; 5’-CCGAAGAGTTTGCGTTGGAACAA  MT-A1-R1; 5’-TTCCCTTTCTCTTCGATTAGGCTGT |
| *MTLA2* (orf19.3200) | MT-A2-F1; 5’-AGAGTCGCAATGGCACGATAAA  MT-A2-R1; 5’-TCTCCGTGGTTTCCTATTCAGCTT |
| *WOR1*  (orf19.4884) | MT-WOR-F1; 5’-catgaacgtgaacgaggcgtgct  MT-WOR-R1; 5’-tcgtactcgtcgtcgggaccaaa |

*Primers used for RT-PCR
