# Supplementary material for: Widespread Occurrence of Dosage Compensation in Candida albicans
Source: PLoS One. 2010 Jun 11;5(6):e10856. doi: 10.1371/journal.pone.0010856 (PMC2883996; doi:10.1371/journal.pone.0010856)
Supplement: Table S2 — Expression arraysa and aCGH values calculated as mean ratios Sor55/3153A for all Ch5b genes. (1.05 MB DOC) [file pone.0010856.s004.doc]

|  |  |  |  |  |  |  |  |  |  |  |  |  |  |
| --- | --- | --- | --- | --- | --- | --- | --- | --- | --- | --- | --- | --- | --- |
| **Table S2.** Expression arrays^a^ and aCGH values calculated as mean ratios Sor55/3153A for all Ch5b genes. | | | | | | | | | |  |  |  |  |
|  |  |  | **Expression arrays** |  |  |  |  |  |  |  |  | **CGH arrays** |  |
|  |  |  | **no backgrd subtracted** | **backgrd subtracted** | | |  |  |  |  |  |  |  |
|  |  | **3153A** | **Sor55** | **3153A** |  | **Sor55** |  | **Sor55** |  |  |  | **Sor55** |  |
|  |  |  |  |  |  |  |  | **3153A** |  |  |  | **3153A** |  |
| **orf** | **Gene** | **Mean** | **Mean** | **Mean** | **± sd** | **Mean** | **± sd** | **Ratio** | **± sd** | **p-value** | **p.paired** | **Ratio** | **± sd** |
| orf19.3198^b^ | *OBPa* | 1446.9 | 282.0 | 1688.3 | 857 | 181.0 | 124 | **0.11** | 0.057 | 3.10E-05 | 0.0001558 | **0.05** | 0.007 |
| orf19.3895 | *CHT2* | 3109.6 | 1300.2 | 3692.0 | 1945 | 915.4 | 362 | **0.25** | 0.234 | 0.0005318 | 0.0059391 | **0.57** | 0.081 |
| orf19.3199 | *PIKa* | 940.6 | 449.7 | 1079.4 | 678 | 282.1 | 168 | **0.26** | 0.171 | 0.0078104 | 0.0013238 | **0.05** | 0.009 |
| orf19.3982 |  | 1040.9 | 520.9 | 1113.7 | 1093 | 333.9 | 140 | **0.3** | 0.303 | 0.0909373 | 0.0301879 | **0.57** | 0.022 |
| orf19.3197 | *PAPa* | 1039.6 | 659.3 | 1190.1 | 307 | 438.5 | 160 | **0.37** | 0.153 | 0.0003301 | 0.0021912 | **0.05** | 0.006 |
| orf19.4235 | *PDE1* | 630.3 | 483.0 | 719.1 | 992 | 327.3 | 98 | **0.46** | 0.989 | 0.6030451 | 0.6176707 | **0.54** | 0.038 |
| orf19.4274 | *PUT1* | 1492.5 | 1367.9 | 2112.2 | 2764 | 1011.9 | 911 | **0.48** | 0.461 | 0.4089916 | 0.0921382 | **0.59** | 0.026 |
| orf19.5685 | *THS1* | 4161.3 | 3810.6 | 4811.3 | 2233 | 2321.7 | 495 | **0.48** | 0.35 | 0.0409204 | 0.0507323 | **0.57** | 0.064 |
| orf19.3981 | *MAL31* | 577.3 | 418.7 | 666.5 | 322 | 324.2 | 249 | **0.49** | 0.264 | 0.0322624 | 0.0187591 | **0.60** | 0.024 |
| orf19.1963 | *GDS1* | 1548.4 | 1396.9 | 1736.6 | 799 | 867.9 | 188 | **0.5** | 0.245 | 0.0380731 | 0.0198302 | **0.57** | 0.026 |
| orf19.1977 | *URA4* | 2488.9 | 2347.5 | 2863.1 | 2070 | 1421.5 | 613 | **0.5** | 0.194 | 0.1128815 | 0.0152272 | **0.59** | 0.042 |
| orf19.3190^b^ | *HAL9c* | 351.9 | 279.8 | 353.4 | 175 | 176.4 | 54 | **0.5** | 0.989 | 0.0878142 | 0.1754267 | **0.34** | 0.009 |
| orf19.941 | *SEC14* | 3119.5 | 3141.3 | 4006.5 | 2191 | 2027.9 | 1106 | **0.51** | 0.049 | 0.0640257 | 1.25E-05 | **0.57** | 0.026 |
| orf19.1956 |  | 1378.2 | 1330.6 | 1551.0 | 993 | 802.7 | 372 | **0.52** | 0.262 | 0.1881144 | 0.0173143 | **0.62** | 0.044 |
| orf19.1960 | *CLN3* | 663.3 | 631.5 | 700.1 | 380 | 363.8 | 197 | **0.52** | 0.145 | 0.0905793 | 0.0042575 | **0.60** | 0.017 |
| orf19.4000 | *PHO2* | 733.2 | 675.9 | 835.7 | 259 | 432.2 | 75 | **0.52** | 0.143 | 0.0048362 | 0.0022086 | **0.61** | 0.058 |
| orf19.4258 | *FIP1* | 1354.8 | 1103.4 | 1474.4 | 1172 | 764.9 | 426 | **0.52** | 0.458 | 0.164558 | 0.1982075 | **0.62** | 0.055 |
| orf19.568 | *SPE2* | 2870.1 | 2944.4 | 3382.5 | 1454 | 1766.2 | 660 | **0.52** | 0.164 | 0.0459865 | 0.0035532 | **0.59** | 0.041 |
| orf19.3893 | *SCW11* | 1875.1 | 1702.9 | 2256.7 | 1424 | 1190.5 | 972 | **0.53** | 0.247 | 0.1427044 | 0.0120401 | **0.65** | 0.097 |
| orf19.4301 |  | 624.5 | 588.6 | 689.5 | 265 | 364.7 | 94 | **0.53** | 0.273 | 0.0102979 | 0.0257948 | **0.55** | 0.036 |
| orf19.1946 |  | 1461.2 | 1444.4 | 1672.7 | 1598 | 907.4 | 701 | **0.54** | 0.823 | 0.616143 | 0.2697636 | **0.58** | 0.023 |
| orf19.3181.1 | *NCE11* | 1343.1 | 1349.1 | 1663.9 | 452 | 897.2 | 222 | **0.54** | 0.201 | 0.0039383 | 0.0072377 | **0.64** | 0.094 |
| orf19.3216 |  | 1219.4 | 1254.9 | 1392.6 | 1121 | 755.8 | 478 | **0.54** | 0.275 | 0.2613672 | 0.0212886 | **0.68** | 0.031 |
| orf19.3954 | *PSD2* | 735.1 | 707.3 | 799.8 | 494 | 435.1 | 165 | **0.54** | 0.35 | 0.1515489 | 0.0361878 | **0.60** | 0.043 |
| orf19.4308 | *HSL1* | 928.2 | 926.1 | 1037.4 | 534 | 564.5 | 292 | **0.54** | 0.194 | 0.1121029 | 0.0088534 | **0.63** | 0.058 |
| orf19.1945 | *AUR1* | 842.2 | 783.5 | 950.7 | 236 | 526.8 | 106 | **0.55** | 0.227 | 0.0015069 | 0.0226085 | **0.61** | 0.032 |
| orf19.6298 | *SBP4* | 372.8 | 324.1 | 381.3 | 170 | 210.5 | 59 | **0.55** | 0.474 | 0.0591684 | 0.1168835 | **0.36** | 0.048 |
| orf19.6665 | *NUP4* | 3088.7 | 3041.5 | 3833.5 | 2242 | 2124.0 | 1748 | **0.55** | 0.238 | 0.1361866 | 0.0099351 | **0.62** | 0.050 |
| orf19.979 | *FAS1* | 4111.8 | 4431.2 | 4948.7 | 2411 | 2698.4 | 613 | **0.55** | 0.439 | 0.1001782 | 0.0575768 | **0.62** | 0.073 |
| orf19.980 | *VIP1* | 2705.5 | 2601.0 | 3277.0 | 1583 | 1792.3 | 979 | **0.55** | 0.284 | 0.0703455 | 0.0153194 | **0.60** | 0.068 |
| orf19.4152 | *EFT3* | 6540.3 | 7097.8 | 7769.7 | 3615 | 4373.4 | 1601 | **0.56** | 0.381 | 0.0728045 | 0.0664729 | **0.59** | 0.029 |
| orf19.3211 | *RCF3* | 1644.3 | 1837.6 | 1869.0 | 608 | 1068.7 | 356 | **0.57** | 0.194 | 0.0187971 | 0.0117588 | **0.59** | 0.026 |
| orf19.3949 | *YTA7* | 859.0 | 868.8 | 973.2 | 301 | 550.6 | 86 | **0.57** | 0.257 | 0.0098703 | 0.0195712 | **0.62** | 0.059 |
| orf19.4136 | *TOF1* | 606.8 | 612.0 | 650.5 | 485 | 372.9 | 208 | **0.57** | 0.778 | 0.4552892 | 0.2136987 | **0.58** | 0.035 |
| orf19.4295 | *HIR2* | 472.3 | 442.8 | 482.4 | 144 | 276.3 | 65 | **0.57** | 0.183 | 0.0061774 | 0.0083908 | **0.60** | 0.030 |
| orf19.6294 | *MYO1* | 531.6 | 521.4 | 558.8 | 207 | 318.6 | 85 | **0.57** | 0.183 | 0.0198146 | 0.0074455 | **0.60** | 0.033 |
| orf19.3964 | *ASH2* | 663.9 | 655.3 | 727.7 | 254 | 423.4 | 148 | **0.58** | 0.169 | 0.0225504 | 0.006291 | **0.65** | 0.080 |
| orf19.4242 | *CST20* | 464.6 | 442.7 | 495.1 | 212 | 286.8 | 48 | **0.58** | 0.455 | 0.0639761 | 0.108711 | **0.56** | 0.029 |
| orf19.4255 | *ECM331* | 6354.1 | 7476.9 | 7372.9 | 6855 | 4308.1 | 3343 | **0.58** | 0.189 | 0.4773198 | 0.0112338 | **0.59** | 0.028 |
| orf19.576 | *CTF8* | 1078.5 | 1303.2 | 1336.4 | 1322 | 775.6 | 651 | **0.58** | 17.99 | 0.8945785 | 0.8608107 | **0.53** | 0.030 |
| orf19.3969 | *HSR1* | 495.2 | 478.8 | 541.1 | 191 | 320.0 | 84 | **0.59** | 0.342 | 0.0529695 | 0.064533 | **0.66** | 0.072 |
| orf19.4257 | *INT1* | 530.8 | 521.7 | 556.1 | 355 | 328.9 | 97 | **0.59** | 0.695 | 0.3213478 | 0.240507 | **0.60** | 0.056 |
| orf19.4335 | *TNA1* | 977.3 | 1058.0 | 1080.8 | 451 | 638.9 | 260 | **0.59** | 0.203 | 0.0804692 | 0.0091701 | **0.55** | 0.031 |
| orf19.4337 | *ESBP6* | 664.7 | 671.1 | 759.2 | 552 | 447.0 | 254 | **0.59** | 0.785 | 0.5000689 | 0.268518 | **0.72** | 0.048 |
| orf19.6660 |  | 2899.4 | 3297.5 | 3749.8 | 952 | 2228.6 | 855 | **0.59** | 0.169 | 0.0165304 | 0.0059294 | **0.59** | 0.043 |
| orf19.4281 | *IFH1* | 827.3 | 842.4 | 934.8 | 321 | 557.2 | 323 | **0.6** | 0.276 | 0.0651275 | 0.0212568 | **0.62** | 0.060 |
| orf19.962 |  | 1186.3 | 1406.7 | 1410.0 | 42 | 852.3 | 174 | **0.6** | 0.129 | 0.042182 | 0.0481284 | **0.58** | 0.067 |
| orf19.1105 |  | 46508.7 | 54014.5 | 57595.6 | 18859 | 35256.9 | 7190 | **0.61** | 0.263 | 0.1359187 | 0.210645 | **0.74** | 0.055 |
| orf19.3942 | *RPL43A* | 34630.9 | 39989.5 | 44137.8 | 22610 | 26704.5 | 19540 | **0.61** | 0.193 | 0.163437 | 0.0171254 | **0.68** | 0.078 |
| orf19.3984 |  | 392.9 | 371.0 | 432.9 | 264 | 265.7 | 126 | **0.61** | 0.866 | 0.4190714 | 0.3829176 | **0.64** | 0.023 |
| orf19.4311 | *YNK1* | 8656.5 | 10973.5 | 10653.9 | 7548 | 6504.8 | 4687 | **0.61** | 0.193 | 0.305484 | 0.0175021 | **0.51** | 0.023 |
| orf19.4312 | *SPT8* | 897.5 | 955.3 | 1014.1 | 502 | 613.8 | 200 | **0.61** | 0.269 | 0.1281087 | 0.0412881 | **0.61** | 0.028 |
| orf19.940 | *BUD2* | 374.6 | 365.0 | 381.6 | 166 | 232.3 | 40 | **0.61** | 0.796 | 0.1773104 | 0.2226814 | **0.61** | 0.029 |
| orf19.1961 |  | 811.3 | 949.5 | 908.9 | 637 | 564.8 | 385 | **0.62** | 0.221 | 0.3325206 | 0.0136866 | **0.55** | 0.047 |
| orf19.4032 | *RPN5* | 1348.7 | 1467.7 | 1580.8 | 289 | 973.5 | 594 | **0.62** | 0.434 | 0.0427183 | 0.0529778 | **0.57** | 0.027 |
| orf19.4265 | *UAP1* | 992.9 | 1118.3 | 1141.1 | 521 | 704.3 | 199 | **0.62** | 0.246 | 0.1079701 | 0.0298366 | **0.60** | 0.037 |
| orf19.1973 | *HAP5* | 739.7 | 796.0 | 834.6 | 258 | 524.1 | 108 | **0.63** | 0.236 | 0.0290261 | 0.0275583 | **0.62** | 0.050 |
| orf19.3912 | *GLN3* | 962.3 | 1041.1 | 1093.5 | 245 | 687.5 | 157 | **0.63** | 0.244 | 0.0062832 | 0.025758 | **0.63** | 0.051 |
| orf19.4003 | *TIP20* | 1169.2 | 1312.1 | 1368.1 | 772 | 867.4 | 304 | **0.63** | 0.352 | 0.1804667 | 0.1247436 | **0.59** | 0.051 |
| orf19.4148 |  | 559.8 | 571.3 | 598.4 | 237 | 379.0 | 82 | **0.63** | 0.295 | 0.0481603 | 0.0713545 | **0.60** | 0.032 |
| orf19.586 | *ERV46* | 1831.2 | 2074.6 | 2153.7 | 870 | 1364.2 | 912 | **0.63** | 0.335 | 0.1424605 | 0.0281594 | **0.58** | 0.047 |
| orf19.3174 | *CDC24* | 889.8 | 983.1 | 1030.7 | 379 | 657.3 | 212 | **0.64** | 0.256 | 0.0526061 | 0.0568131 | **0.56** | 0.027 |
| orf19.3997 | *ADH1* | 45242.7 | 55286.1 | 61178.1 | 27532 | 39205.5 | 17001 | **0.64** | 0.184 | 0.1581824 | 0.0208556 | **0.72** | 0.081 |
| orf19.4023 | *MRP2* | 1825.4 | 2180.4 | 2273.7 | 443 | 1462.2 | 609 | **0.64** | 0.194 | 0.0281468 | 0.0175035 | **0.60** | 0.032 |
| orf19.4297 | *CKB22* | 789.9 | 863.6 | 913.0 | 190 | 584.1 | 201 | **0.64** | 0.33 | 0.0155509 | 0.0495753 | **0.59** | 0.033 |
| orf19.976 | *BRE1* | 938.8 | 1042.2 | 1058.3 | 871 | 674.8 | 536 | **0.64** | 0.55 | 0.5221911 | 0.1748181 | **0.58** | 0.031 |
| orf19.3941 | *URA7* | 2248.4 | 2831.3 | 2717.2 | 690 | 1776.5 | 406 | **0.65** | 0.18 | 0.0116423 | 0.0106626 | **0.60** | 0.024 |
| orf19.3944 | *GRR1* | 1131.6 | 1258.4 | 1316.8 | 459 | 855.4 | 351 | **0.65** | 0.377 | 0.0815292 | 0.0880295 | **0.59** | 0.041 |
| orf19.4149 | *RPS11A* | 25318.3 | 32098.7 | 32710.7 | 25918 | 21363.3 | 17294 | **0.65** | 0.228 | 0.4819307 | 0.0286533 | **0.68** | 0.032 |
| orf19.4322 | *DAP2* | 451.5 | 464.4 | 478.7 | 289 | 313.2 | 150 | **0.65** | 1.008 | 0.5684437 | 0.4273898 | **0.62** | 0.038 |
| orf19.6676 | *DPH5* | 2785.4 | 3409.8 | 3356.0 | 1746 | 2182.1 | 720 | **0.65** | 0.232 | 0.1837565 | 0.0631896 | **0.62** | 0.093 |
| orf19.922 | *ERG11* | 1702.6 | 1965.3 | 2004.0 | 455 | 1300.0 | 370 | **0.65** | 0.318 | 0.0161139 | 0.0778533 | **0.56** | 0.026 |
| orf19.964 | *SMC4* | 997.8 | 1182.9 | 1116.0 | 654 | 728.1 | 427 | **0.65** | 0.17 | 0.2581951 | 0.0141482 | **0.62** | 0.056 |
| orf19.969 | *PRS2* | 5186.1 | 6290.9 | 6218.9 | 1797 | 4060.9 | 856 | **0.65** | 0.391 | 0.0368528 | 0.1073094 | **0.61** | 0.073 |
| orf19.3192 | *STI1* | 1586.2 | 2211.7 | 2042.1 | 1013 | 1350.8 | 346 | **0.66** | 0.293 | 0.1328933 | 0.1051728 | **0.53** | 0.025 |
| orf19.3911 | *SAH1* | 9554.9 | 12344.6 | 12258.8 | 1908 | 8148.0 | 2691 | **0.66** | 0.217 | 0.027054 | 0.0295055 | **0.58** | 0.040 |
| orf19.3938 |  | 781.1 | 902.5 | 847.1 | 416 | 557.2 | 341 | **0.66** | 0.337 | 0.1965211 | 0.0419279 | **0.57** | 0.054 |
| orf19.4040 | *ILV3* | 3813.1 | 5045.8 | 4568.1 | 4472 | 3021.9 | 2799 | **0.66** | 0.251 | 0.7630381 | 0.2048648 | **0.63** | 0.088 |
| orf19.4150 |  | 700.4 | 736.0 | 781.4 | 529 | 513.2 | 415 | **0.66** | 0.681 | 0.4983706 | 0.2762118 | **0.61** | 0.029 |
| orf19.4262 | *JSN1* | 578.5 | 655.0 | 611.0 | 241 | 405.3 | 99 | **0.66** | 0.216 | 0.0844293 | 0.0360974 | **0.61** | 0.041 |
| orf19.4324^b^ |  | 271.4 | 269.5 | 250.3 | 184 | 165.5 | 41 | **0.66** | 1.593 | 0.7997936 | 0.82438 | **0.61** | 0.036 |
| orf19.1287 |  | 2475.9 | 3137.8 | 2965.1 | 1481 | 1987.7 | 832 | **0.67** | 0.217 | 0.2870005 | 0.0372324 | **0.63** | 0.049 |
| orf19.1959 |  | 565.3 | 655.7 | 634.1 | 364 | 425.6 | 128 | **0.67** | 1.022 | 0.4873363 | 0.4385175 | **0.59** | 0.041 |
| orf19.4035 | *GAS1* | 3943.9 | 4583.8 | 4770.5 | 1244 | 3204.7 | 1440 | **0.67** | 0.466 | 0.0583707 | 0.1340091 | **0.64** | 0.103 |
| orf19.4318 | *MIG1* | 1334.3 | 1599.5 | 1597.8 | 518 | 1066.7 | 315 | **0.67** | 0.223 | 0.0512201 | 0.0401983 | **0.60** | 0.028 |
| orf19.5683 |  | 1079.3 | 1287.3 | 1382.0 | 665 | 924.5 | 842 | **0.67** | 0.294 | 0.1350688 | 0.07469 | **0.56** | 0.013 |
| orf19.5698 | *RPL1* | 1175.0 | 1526.2 | 1407.4 | 353 | 948.0 | 389 | **0.67** | 0.3 | 0.0832455 | 0.0394298 | **0.63** | 0.075 |
| orf19.587 | *TEX1* | 586.6 | 690.6 | 633.5 | 185 | 425.0 | 83 | **0.67** | 0.165 | 0.0359451 | 0.0113998 | **0.68** | 0.071 |
| orf19.4142 |  | 551.4 | 628.8 | 619.0 | 199 | 421.4 | 144 | **0.68** | 0.467 | 0.1278519 | 0.143234 | **0.57** | 0.028 |
| orf19.4278 |  | 824.2 | 995.9 | 948.1 | 250 | 647.4 | 155 | **0.68** | 0.23 | 0.0395133 | 0.03269 | **0.60** | 0.048 |
| orf19.4282 |  | 644.4 | 761.2 | 706.1 | 381 | 483.3 | 224 | **0.68** | 0.33 | 0.3877093 | 0.1046198 | **0.62** | 0.036 |
| orf19.5693 | *GAA1* | 577.7 | 654.9 | 609.3 | 338 | 414.3 | 149 | **0.68** | 0.487 | 0.3158962 | 0.1822387 | **0.65** | 0.092 |
| orf19.945 | *DID4* | 742.2 | 885.7 | 856.2 | 234 | 581.4 | 136 | **0.68** | 0.186 | 0.0346405 | 0.0322865 | **0.47** | 0.015 |
| orf19.966 | *DUS4* | 841.5 | 960.6 | 984.5 | 612 | 671.5 | 461 | **0.68** | 0.512 | 0.4316292 | 0.2065161 | **0.62** | 0.063 |
| orf19.3160 | *HSP13* | 36963.5 | 48761.5 | 47543.4 | 24400 | 32936.6 | 16687 | **0.69** | 0.269 | 0.2745433 | 0.1170741 |  |  |
| orf19.3166 | *DOP1* | 610.9 | 738.4 | 668.6 | 237 | 462.0 | 133 | **0.69** | 0.202 | 0.1515814 | 0.0315142 | **0.60** | 0.065 |
| orf19.3177 | *RIB2* | 1508.3 | 1868.0 | 1768.6 | 571 | 1224.1 | 274 | **0.69** | 0.282 | 0.0596457 | 0.081185 | **0.58** | 0.064 |
| orf19.3915 |  | 4087.6 | 4997.1 | 4839.7 | 2689 | 3362.8 | 1283 | **0.69** | 0.473 | 0.2451219 | 0.2726435 | **0.60** | 0.049 |
| orf19.3994 | *OST3* | 849.3 | 1076.8 | 984.8 | 302 | 676.7 | 197 | **0.69** | 0.136 | 0.0629593 | 0.005429 | **0.59** | 0.037 |
| orf19.4030 | *PRI1* | 1281.1 | 1708.8 | 1494.3 | 593 | 1031.3 | 323 | **0.69** | 0.44 | 0.1227141 | 0.1320905 | **0.60** | 0.046 |
| orf19.4036 | *APM1* | 730.4 | 907.1 | 843.3 | 313 | 582.6 | 90 | **0.69** | 0.389 | 0.0949747 | 0.1641428 | **0.61** | 0.040 |
| orf19.4219 | *VCP1* | 770.3 | 986.2 | 862.7 | 532 | 593.2 | 266 | **0.69** | 0.483 | 0.4560654 | 0.2002117 | **0.64** | 0.115 |
| orf19.4225 | *LEU3* | 852.2 | 1039.0 | 955.3 | 658 | 659.7 | 399 | **0.69** | 0.39 | 0.5386392 | 0.1368682 | **0.64** | 0.072 |
| orf19.4240 | *PER1* | 788.0 | 962.4 | 885.7 | 128 | 612.4 | 176 | **0.69** | 0.198 | 0.0339117 | 0.0245924 | **0.60** | 0.071 |
| orf19.5680 |  | 895.5 | 1018.8 | 1051.5 | 288 | 729.6 | 471 | **0.69** | 0.29 | 0.0951342 | 0.0434082 | **0.61** | 0.068 |
| orf19.579 | *FOL1* | 682.1 | 836.9 | 753.1 | 357 | 522.7 | 139 | **0.69** | 0.276 | 0.2355859 | 0.1122886 | **0.63** | 0.030 |
| orf19.585 | *MRPL17* | 903.1 | 1094.0 | 1019.4 | 376 | 705.4 | 163 | **0.69** | 0.402 | 0.1601619 | 0.178364 | **0.57** | 0.055 |
| orf19.1115 | *GUK1* | 909.0 | 1147.9 | 1032.6 | 490 | 723.1 | 391 | **0.7** | 0.132 | 0.2798909 | 0.0035849 | **0.50** | 0.029 |
| orf19.1942 | *AZR2* | 823.6 | 957.1 | 991.0 | 366 | 692.7 | 448 | **0.7** | 0.242 | 0.1121137 | 0.0339346 | **0.65** | 0.040 |
| orf19.3974 | *PUT2* | 1952.7 | 2614.3 | 2382.4 | 868 | 1661.3 | 1044 | **0.7** | 0.237 | 0.1678253 | 0.0174657 | **0.61** | 0.039 |
| orf19.3980 | *SLH1* | 554.4 | 635.2 | 594.8 | 298 | 414.8 | 139 | **0.7** | 0.315 | 0.2773815 | 0.1458354 | **0.62** | 0.053 |
| orf19.3996 | *GPI10* | 649.0 | 765.2 | 713.7 | 215 | 500.7 | 95 | **0.7** | 0.25 | 0.0617227 | 0.0531901 | **0.55** | 0.024 |
| orf19.3999 |  | 316.7 | 333.3 | 298.3 | 179 | 207.8 | 52 | **0.7** | 2.264 | 0.7893132 | 0.7884274 | **0.64** | 0.034 |
| orf19.4004 | *CCT3* | 2221.6 | 2928.8 | 2652.3 | 1764 | 1854.1 | 1011 | **0.7** | 0.315 | 0.5336111 | 0.1423493 | **0.60** | 0.031 |
| orf19.4016 | *COR1* | 5142.1 | 6953.8 | 6409.8 | 2860 | 4475.2 | 1093 | **0.7** | 0.171 | 0.1607258 | 0.0299984 | **0.60** | 0.066 |
| orf19.4280 |  | 349.7 | 368.4 | 379.3 | 262 | 264.2 | 140 | **0.7** | 0.678 | 0.7489263 | 0.6463621 | **0.61** | 0.052 |
| orf19.4346 | *SEC16* | 530.7 | 625.7 | 570.8 | 147 | 397.5 | 88 | **0.7** | 0.271 | 0.0314314 | 0.0634501 | **0.65** | 0.088 |
| orf19.4348 | *RGR1* | 475.2 | 537.1 | 486.8 | 218 | 339.9 | 113 | **0.7** | 0.418 | 0.149646 | 0.1791323 | **0.62** | 0.027 |
| orf19.5689 | *SEC28* | 1181.5 | 1456.1 | 1434.7 | 703 | 1004.1 | 576 | **0.7** | 0.261 | 0.2844568 | 0.0643051 | **0.55** | 0.040 |
| orf19.6663 | *RPS25B* | 14791.1 | 20717.4 | 18663.9 | 6970 | 13096.0 | 4668 | **0.7** | 0.145 | 0.1694671 | 0.0099509 | **0.60** | 0.063 |
| orf19.926 | *EXO1* | 588.0 | 722.6 | 623.7 | 437 | 438.6 | 187 | **0.7** | 0.906 | 0.6302763 | 0.4705032 | **0.58** | 0.026 |
| orf19.931 |  | 712.6 | 822.3 | 797.3 | 287 | 558.5 | 206 | **0.7** | 0.286 | 0.1486581 | 0.1068458 | **0.57** | 0.056 |
| orf19.956 | *CSF1* | 400.4 | 452.2 | 409.8 | 227 | 286.7 | 75 | **0.7** | 1.088 | 0.5678285 | 0.5424937 | **0.58** | 0.042 |
| orf19.1933 | *PEX29* | 548.0 | 646.0 | 578.8 | 184 | 412.0 | 81 | **0.71** | 0.237 | 0.067545 | 0.0843022 | **0.59** | 0.032 |
| orf19.1948 |  | 569.3 | 671.9 | 621.6 | 211 | 438.3 | 160 | **0.71** | 0.304 | 0.1184972 | 0.0972036 | **0.61** | 0.030 |
| orf19.2639 | *LSM7* | 777.0 | 951.6 | 875.2 | 652 | 622.5 | 557 | **0.71** | 0.834 | 0.5371122 | 0.2913102 | **0.57** | 0.056 |
| orf19.3978 |  | 426.0 | 501.7 | 460.2 | 199 | 327.6 | 56 | **0.71** | 0.449 | 0.2571288 | 0.2509547 | **0.59** | 0.053 |
| orf19.4010 | *PAN3* | 470.2 | 539.6 | 509.3 | 179 | 363.3 | 88 | **0.71** | 0.459 | 0.1652209 | 0.2478192 | **0.63** | 0.040 |
| orf19.4294 | *CYC2* | 525.5 | 611.0 | 561.4 | 316 | 397.3 | 159 | **0.71** | 0.94 | 0.5090126 | 0.4202412 | **0.59** | 0.029 |
| orf19.583 | *BNA2* | 3219.2 | 3999.4 | 4102.7 | 2618 | 2908.4 | 3265 | **0.71** | 0.365 | 0.2931321 | 0.0303429 | **0.58** | 0.046 |
| orf19.1283 | *MEC1* | 662.5 | 857.7 | 747.9 | 230 | 535.4 | 117 | **0.72** | 0.353 | 0.079821 | 0.1294839 | **0.61** | 0.042 |
| orf19.3937 | *SDF1* | 366.5 | 415.2 | 370.0 | 247 | 269.7 | 130 | **0.72** | 5.494 | 0.6619456 | 0.6394345 | **0.53** | 0.035 |
| orf19.3960 | *HYS2* | 941.5 | 1247.1 | 1079.2 | 307 | 777.4 | 253 | **0.72** | 0.289 | 0.1009498 | 0.0683084 | **0.62** | 0.052 |
| orf19.4051 | *HTS1* | 2835.1 | 3960.2 | 3460.9 | 642 | 2480.1 | 504 | **0.72** | 0.216 | 0.0206491 | 0.0571422 | **0.63** | 0.091 |
| orf19.4268 |  | 1479.0 | 2056.4 | 1762.5 | 534 | 1267.5 | 764 | **0.72** | 0.264 | 0.1638383 | 0.0253262 | **0.60** | 0.033 |
| orf19.939 | *NAM7* | 1147.1 | 1494.4 | 1358.7 | 601 | 981.1 | 546 | **0.72** | 0.167 | 0.2644672 | 0.012583 | **0.58** | 0.038 |
| orf19.1279 | *CDS1* | 2302.8 | 2975.2 | 2961.6 | 1805 | 2169.2 | 1671 | **0.73** | 0.261 | 0.4613737 | 0.0909016 | **0.60** | 0.024 |
| orf19.1941 | *NUF2* | 517.3 | 617.1 | 559.4 | 160 | 407.7 | 144 | **0.73** | 0.395 | 0.1434092 | 0.1730874 | **0.60** | 0.042 |
| orf19.3167 | *COX10* | 1704.2 | 2193.8 | 1999.8 | 1319 | 1464.7 | 1046 | **0.73** | 0.26 | 0.4698817 | 0.062092 | **0.58** | 0.053 |
| orf19.3201^b^ | *MATa1* | 236.1 | 247.0 | 211.7 | 166 | 157.5 | 23 | **0.73** | 3.307 | 0.5556847 | 0.5691045 | **0.05** | 0.008 |
| orf19.3219 |  | 629.3 | 757.9 | 703.9 | 236 | 514.6 | 221 | **0.73** | 0.32 | 0.1644683 | 0.1416538 | **0.67** | 0.025 |
| orf19.3914 | *CDC332* | 1352.1 | 1674.5 | 1617.0 | 732 | 1178.4 | 744 | **0.73** | 0.413 | 0.3157402 | 0.15569 | **0.56** | 0.033 |
| orf19.4279 | *MNN1* | 2932.2 | 4324.7 | 3697.3 | 1146 | 2704.1 | 362 | **0.73** | 0.204 | 0.1052577 | 0.0327928 | **0.62** | 0.057 |
| orf19.4283 |  | 9604.7 | 14992.9 | 13677.6 | 18874 | 9923.4 | 10523 | **0.73** | 0.43 | 0.7208665 | 0.124987 | **0.61** | 0.027 |
| orf19.4336 | *RPS5* | 11355.9 | 16978.4 | 13716.1 | 6120 | 10037.9 | 2977 | **0.73** | 0.307 | 0.3479115 | 0.205718 | **0.57** | 0.019 |
| orf19.4341 | *CDC91* | 1108.1 | 1476.9 | 1235.9 | 1088 | 901.4 | 772 | **0.73** | 0.366 | 0.6087265 | 0.1103945 | **0.57** | 0.031 |
| orf19.978 | *BDF1* | 527.5 | 644.6 | 561.6 | 297 | 412.4 | 70 | **0.73** | 0.372 | 0.4052031 | 0.3023731 | **0.58** | 0.037 |
| orf19.1936 | *SNF1* | 1792.1 | 2279.9 | 2207.6 | 679 | 1627.7 | 770 | **0.74** | 0.573 | 0.1577941 | 0.2091668 | **0.61** | 0.056 |
| orf19.2644 | *QCR2* | 9646.4 | 13686.4 | 11992.0 | 2989 | 8867.1 | 4184 | **0.74** | 0.279 | 0.1316954 | 0.0563527 | **0.58** | 0.029 |
| orf19.2654 | *RMS1* | 440.9 | 541.2 | 454.0 | 215 | 335.4 | 96 | **0.74** | 0.85 | 0.4531403 | 0.443306 | **0.52** | 0.030 |
| orf19.3175 | *ETF1* | 2522.1 | 3386.5 | 3109.4 | 1223 | 2295.9 | 1260 | **0.74** | 0.172 | 0.2267964 | 0.0145269 | **0.55** | 0.045 |
| orf19.5692 |  | 590.0 | 774.4 | 677.4 | 225 | 502.1 | 90 | **0.74** | 0.636 | 0.2381762 | 0.3435701 | **0.69** | 0.135 |
| orf19.6670 | *CAC2* | 552.7 | 689.2 | 612.2 | 245 | 451.5 | 117 | **0.74** | 0.369 | 0.2287524 | 0.2253605 | **0.59** | 0.031 |
| orf19.949 |  | 823.5 | 1079.3 | 974.2 | 648 | 725.7 | 525 | **0.74** | 0.22 | 0.4986245 | 0.0530348 | **0.57** | 0.042 |
| orf19.1116 |  | 628.2 | 827.2 | 695.1 | 39 | 518.6 | 42 | **0.75** | 0.062 | 0.0096337 | 0.0247202 | **0.66** | 0.076 |
| orf19.2647 |  | 406.1 | 532.3 | 418.2 | 213 | 312.1 | 130 | **0.75** | 0.443 | 0.3927 | 0.2531681 | **0.63** | 0.023 |
| orf19.3193 | *YAP3* | 883.4 | 1154.7 | 1090.4 | 840 | 813.5 | 691 | **0.75** | 0.57 | 0.5858643 | 0.2591821 | **0.55** | 0.054 |
| orf19.3207 | *CCN1* | 360.7 | 433.5 | 345.9 | 160 | 258.0 | 93 | **0.75** | 0.465 | 0.2859517 | 0.222075 | **0.59** | 0.036 |
| orf19.3950 | *MSM1* | 3597.2 | 5828.5 | 4997.8 | 6463 | 3741.7 | 3568 | **0.75** | 0.507 | 0.7733621 | 0.4223501 | **0.62** | 0.063 |
| orf19.3976 | *JNM1* | 929.4 | 1277.5 | 1121.6 | 1123 | 841.2 | 790 | **0.75** | 1.996 | 0.7384998 | 0.6448258 | **0.60** | 0.027 |
| orf19.4048 | *DES1* | 3414.4 | 4626.1 | 4229.7 | 1946 | 3177.3 | 2353 | **0.75** | 0.338 | 0.286614 | 0.1056181 | **0.62** | 0.073 |
| orf19.567 | *TFB3* | 516.7 | 634.1 | 602.2 | 487 | 453.1 | 351 | **0.75** | 1.085 | 0.7622619 | 0.6244231 | **0.61** | 0.025 |
| orf19.6662 |  | 463.3 | 606.7 | 511.5 | 200 | 381.9 | 38 | **0.75** | 0.524 | 0.4749773 | 0.5412656 | **0.58** | 0.027 |
| orf19.923 | *THR1* | 798.5 | 1080.2 | 870.6 | 364 | 651.7 | 295 | **0.75** | 0.207 | 0.2836668 | 0.0425016 | **0.60** | 0.066 |
| orf19.1971 |  | 979.4 | 1271.8 | 1155.0 | 1054 | 873.4 | 894 | **0.76** | 27.91 | 0.6916269 | 0.614589 | **0.61** | 0.039 |
| orf19.3900 | *UGO1* | 816.0 | 1051.0 | 918.7 | 313 | 699.4 | 186 | **0.76** | 0.334 | 0.1888203 | 0.1806509 | **0.66** | 0.061 |
| orf19.3955 | *MES1* | 1188.6 | 1709.8 | 1380.4 | 675 | 1051.1 | 457 | **0.76** | 0.24 | 0.4380766 | 0.0894419 | **0.58** | 0.042 |
| orf19.3959 | *SSD1* | 1146.8 | 1555.8 | 1340.7 | 392 | 1021.9 | 437 | **0.76** | 0.301 | 0.226068 | 0.1193568 | **0.62** | 0.055 |
| orf19.4022 | *SDH5* | 871.2 | 1212.9 | 1033.9 | 430 | 787.9 | 284 | **0.76** | 0.181 | 0.2323107 | 0.0232762 | **0.62** | 0.048 |
| orf19.4041 | *PEX4* | 2081.4 | 2811.2 | 2859.0 | 2362 | 2168.2 | 1757 | **0.76** | 0.428 | 0.6107684 | 0.2442902 | **0.70** | 0.121 |
| orf19.4317 | *GRE3* | 994.6 | 1445.5 | 1190.5 | 454 | 905.3 | 354 | **0.76** | 0.19 | 0.2695184 | 0.037387 | **0.60** | 0.044 |
| orf19.947 | *MRP17* | 503.1 | 677.5 | 527.0 | 251 | 402.4 | 147 | **0.76** | 0.309 | 0.3941122 | 0.1500571 | **0.55** | 0.021 |
| orf19.974 | *ROT2* | 975.7 | 1230.0 | 1104.5 | 245 | 838.2 | 243 | **0.76** | 0.389 | 0.1138904 | 0.1866653 | **0.58** | 0.050 |
| orf19.1285 |  | 871.7 | 1166.0 | 1017.0 | 746 | 781.5 | 593 | **0.77** | 0.66 | 0.7217241 | 0.4035707 | **0.56** | 0.037 |
| orf19.1958^b^ |  | 190.0 | 205.2 | 149.8 | 193 | 11.0 | 32 | **0.77** | 1.981 | 0.585295 | 0.6076452 | **0.73** | 0.044 |
| orf19.1966 | *BUD23* | 2517.7 | 3788.1 | 3464.6 | 4355 | 2679.4 | 2584 | **0.77** | 0.295 | 0.8704149 | 0.4402686 | **0.61** | 0.032 |
| orf19.3188 | *HAL9a* | 568.2 | 777.2 | 626.9 | 497 | 485.2 | 335 | **0.77** | 2.509 | 0.9962881 | 0.9940596 | **0.58** | 0.056 |
| orf19.4002 | *DUN1* | 334.4 | 400.3 | 327.9 | 145 | 251.6 | 58 | **0.77** | 0.471 | 0.3899052 | 0.3525271 | **0.56** | 0.026 |
| orf19.4131 |  | 1802.7 | 2506.1 | 2165.5 | 1341 | 1673.8 | 1092 | **0.77** | 0.35 | 0.570533 | 0.2391354 | **0.61** | 0.017 |
| orf19.4145 | *HAP1* | 485.4 | 615.0 | 514.0 | 154 | 394.7 | 65 | **0.77** | 0.328 | 0.159778 | 0.1953829 | **0.55** | 0.026 |
| orf19.4347 | *PRR1* | 1072.1 | 1279.7 | 1292.3 | 489 | 995.9 | 669 | **0.77** | 0.512 | 0.265155 | 0.2771998 | **0.57** | 0.060 |
| orf19.6295 | *MAS2* | 938.5 | 1278.1 | 1071.5 | 893 | 826.3 | 751 | **0.77** | 0.575 | 0.7393888 | 0.3344621 | **0.58** | 0.028 |
| orf19.6297 | *DEG1* | 402.4 | 524.0 | 424.0 | 172 | 326.5 | 79 | **0.77** | 0.766 | 0.3865256 | 0.5010768 | **0.58** | 0.022 |
| orf19.951 |  | 509.6 | 616.6 | 595.8 | 333 | 460.1 | 334 | **0.77** | 0.198 | 0.5837395 | 0.2069599 | **0.64** | 0.055 |
| orf19.970 |  | 436.1 | 555.2 | 456.7 | 142 | 353.9 | 8 | **0.77** | 0.263 | 0.3573634 | 0.3517012 | **0.59** | 0.032 |
| orf19.3218 |  | 409.4 | 510.0 | 424.1 | 174 | 331.5 | 118 | **0.78** | 0.419 | 0.3740478 | 0.3055572 | **0.68** | 0.051 |
| orf19.3931 | *SFC1* | 881.6 | 1179.5 | 1005.2 | 683 | 787.0 | 524 | **0.78** | 0.495 | 0.6372938 | 0.3668637 | **0.66** | 0.072 |
| orf19.3946 | *COX18* | 543.9 | 671.6 | 580.4 | 354 | 454.5 | 235 | **0.78** | 0.801 | 0.7963794 | 0.6849578 | **0.61** | 0.065 |
| orf19.3972 | *GOT1* | 739.2 | 1002.7 | 835.4 | 559 | 653.8 | 432 | **0.78** | 0.523 | 0.7023913 | 0.4184869 | **0.60** | 0.061 |
| orf19.4009 | *CNB1* | 1399.6 | 2126.8 | 1668.5 | 232 | 1298.0 | 237 | **0.78** | 0.187 | 0.0219101 | 0.0503399 | **0.61** | 0.032 |
| orf19.4233 | *THR4* | 2373.3 | 3432.5 | 2912.7 | 2076 | 2264.0 | 1681 | **0.78** | 0.217 | 0.6596701 | 0.0678364 | **0.63** | 0.060 |
| orf19.577 |  | 1088.3 | 1561.4 | 1308.2 | 446 | 1014.2 | 262 | **0.78** | 0.142 | 0.2237276 | 0.0240519 | **0.62** | 0.044 |
| orf19.930 | *PET9* | 25355.2 | 37508.8 | 32717.8 | 16822 | 25378.0 | 16760 | **0.78** | 0.155 | 0.3722509 | 0.0111162 | **0.60** | 0.025 |
| orf19.967 | *NUC1* | 1276.9 | 1941.3 | 1522.1 | 429 | 1188.2 | 271 | **0.78** | 0.319 | 0.1589602 | 0.1410898 | **0.59** | 0.072 |
| orf19.1108 | *HAM1* | 2004.1 | 3008.7 | 2548.8 | 1123 | 2010.5 | 870 | **0.79** | 0.171 | 0.3719833 | 0.0381669 | **0.60** | 0.032 |
| orf19.1968.1 |  | 450.0 | 579.1 | 481.9 | 153 | 379.6 | 85 | **0.79** | 0.546 | 0.4327212 | 0.580971 | **0.72** | 0.048 |
| orf19.3180 | *INP54* | 357.1 | 429.0 | 362.5 | 186 | 285.0 | 148 | **0.79** | 1.014 | 0.6421556 | 0.5999228 | **0.59** | 0.066 |
| orf19.3962 | *HAS1* | 2885.0 | 4154.9 | 3655.1 | 512 | 2883.1 | 1126 | **0.79** | 0.275 | 0.132361 | 0.0750719 | **0.62** | 0.048 |
| orf19.4021 |  | 1026.7 | 1509.5 | 1211.7 | 155 | 960.9 | 85 | **0.79** | 0.133 | 0.007789 | 0.0204267 | **0.66** | 0.051 |
| orf19.5684 | *MRPL14* | 1530.4 | 2292.6 | 1959.7 | 681 | 1552.4 | 521 | **0.79** | 0.203 | 0.2742648 | 0.0688138 | **0.53** | 0.063 |
| orf19.928 |  | 312.4 | 373.0 | 304.4 | 232 | 245.5 | 96 | **0.79** | 2.632 | 0.5970895 | 0.5941538 | **0.61** | 0.025 |
| orf19.1105.2 |  | 15249.5 | 21151.9 | 26157.4 | 43788 | 20993.5 | 31482 | **0.8** | 0.91 | 0.941964 | 0.6785542 | **0.59** | 0.027 |
| orf19.1935 |  | 560.5 | 728.4 | 611.0 | 112 | 488.2 | 94 | **0.8** | 0.255 | 0.2203024 | 0.376756 | **0.55** | 0.020 |
| orf19.3182 | *GIS2* | 7491.6 | 11313.1 | 9576.7 | 1679 | 7649.9 | 3059 | **0.8** | 0.227 | 0.179367 | 0.0851993 | **0.66** | 0.153 |
| orf19.3185 | *NAT1* | 1375.7 | 2002.8 | 1610.9 | 476 | 1283.9 | 126 | **0.8** | 0.31 | 0.209012 | 0.2450531 | **0.58** | 0.050 |
| orf19.3940 |  | 286.3 | 351.4 | 274.2 | 158 | 220.1 | 104 | **0.8** | 2.391 | 0.8358599 | 0.873982 | **0.48** | 0.023 |
| orf19.3957 | *FOL2* | 761.5 | 1063.3 | 888.4 | 675 | 709.6 | 554 | **0.8** | 0.57 | 0.7676357 | 0.4363334 | **0.61** | 0.043 |
| orf19.4236 | *RET2* | 859.4 | 1233.9 | 966.6 | 546 | 769.6 | 417 | **0.8** | 0.369 | 0.5652674 | 0.223919 | **0.56** | 0.026 |
| orf19.4342 |  | 694.9 | 988.2 | 845.4 | 649 | 676.3 | 461 | **0.8** | 0.597 | 0.7801765 | 0.5472889 | **0.64** | 0.028 |
| orf19.920 | *RMT2* | 412.9 | 552.4 | 427.7 | 226 | 341.7 | 126 | **0.8** | 1.052 | 0.7522642 | 0.7290819 | **0.57** | 0.027 |
| orf19.1114 |  | 1539.7 | 2346.6 | 1830.9 | 171 | 1485.3 | 281 | **0.81** | 0.217 | 0.1913183 | 0.32152 | **0.54** | 0.047 |
| orf19.1949 | *VPS1* | 2536.9 | 3701.8 | 3016.1 | 862 | 2432.1 | 587 | **0.81** | 0.431 | 0.2446612 | 0.3584035 | **0.67** | 0.097 |
| orf19.1964 |  | 485.5 | 649.0 | 548.0 | 376 | 441.9 | 229 | **0.81** | 0.673 | 0.7459103 | 0.6391725 | **0.62** | 0.025 |
| orf19.1972 | *SET5* | 990.0 | 1337.0 | 1182.2 | 322 | 954.7 | 383 | **0.81** | 0.368 | 0.2920172 | 0.2711371 | **0.64** | 0.051 |
| orf19.2649 | *PCL1* | 946.0 | 1363.8 | 1102.8 | 354 | 896.2 | 411 | **0.81** | 0.323 | 0.340125 | 0.1404966 | **0.60** | 0.028 |
| orf19.3170 | *BUD27* | 548.7 | 739.5 | 603.2 | 232 | 486.5 | 69 | **0.81** | 0.446 | 0.3732125 | 0.4745002 | **0.57** | 0.052 |
| orf19.3183 |  | 488.4 | 714.3 | 570.2 | 336 | 462.4 | 34 | **0.81** | 0.94 | 0.9114682 | 0.9127804 | **0.66** | 0.051 |
| orf19.4288 | *STB4* | 726.1 | 961.0 | 830.8 | 438 | 670.5 | 462 | **0.81** | 0.271 | 0.4839073 | 0.1621987 | **0.61** | 0.037 |
| orf19.4325 |  | 1093.8 | 1536.0 | 1324.6 | 291 | 1074.4 | 340 | **0.81** | 0.385 | 0.2247099 | 0.2361378 | **0.57** | 0.031 |
| orf19.6654 |  | 1155.9 | 1614.8 | 1402.8 | 787 | 1135.5 | 700 | **0.81** | 0.395 | 0.504289 | 0.2364509 | **0.61** | 0.061 |
| orf19.6680 | *FGR27* | 526.1 | 656.3 | 572.4 | 166 | 463.0 | 199 | **0.81** | 0.336 | 0.2677012 | 0.268064 | **0.62** | 0.050 |
| orf19.952 |  | 302.8 | 366.2 | 288.3 | 157 | 232.2 | 51 | **0.81** | 1.06 | 0.7679509 | 0.811047 | **0.60** | 0.032 |
| orf19.953.1 | *COF1* | 10671.4 | 16034.7 | 13926.7 | 3541 | 11337.9 | 5032 | **0.81** | 0.276 | 0.2775489 | 0.1193009 | **0.60** | 0.029 |
| orf19.1934 | *HST3* | 373.5 | 485.7 | 377.9 | 168 | 311.5 | 88 | **0.82** | 0.668 | 0.6789964 | 0.6588026 | **0.50** | 0.020 |
| orf19.3205 | *MRPL36* | 517.0 | 738.3 | 535.6 | 240 | 437.4 | 159 | **0.82** | 0.357 | 0.4918875 | 0.2555158 | **0.60** | 0.046 |
| orf19.3919 |  | 779.6 | 1119.6 | 878.3 | 345 | 716.8 | 267 | **0.82** | 0.206 | 0.4421831 | 0.1243312 | **0.59** | 0.043 |
| orf19.3951 | *YIP1* | 847.1 | 1195.0 | 984.9 | 320 | 810.8 | 349 | **0.82** | 0.275 | 0.3386742 | 0.1720879 | **0.62** | 0.040 |
| orf19.4013 |  | 417.0 | 550.6 | 435.9 | 265 | 357.2 | 152 | **0.82** | 4.487 | 0.8893248 | 0.8797954 | **0.59** | 0.046 |
| orf19.4031 |  | 403.0 | 507.8 | 430.3 | 221 | 354.4 | 160 | **0.82** | 0.831 | 0.7415709 | 0.7286185 | **0.65** | 0.112 |
| orf19.932 | *DNF1* | 563.3 | 813.6 | 619.3 | 194 | 509.6 | 120 | **0.82** | 0.419 | 0.299044 | 0.3548578 | **0.66** | 0.072 |
| orf19.943 | *FET33* | 365.6 | 515.8 | 376.5 | 184 | 310.1 | 87 | **0.82** | 0.944 | 0.7201735 | 0.7593868 | **0.55** | 0.006 |
| orf19.968 | *PGA14* | 9983.9 | 15923.6 | 13551.4 | 6518 | 11061.2 | 5110 | **0.82** | 0.226 | 0.3938828 | 0.1183598 | **0.55** | 0.046 |
| orf19.1967 | *IMG1* | 865.0 | 1327.0 | 975.4 | 961 | 808.6 | 721 | **0.83** | 4.673 | 0.6472379 | 0.5433757 | **0.61** | 0.037 |
| orf19.1974 | *TFS1* | 2602.1 | 4056.7 | 3256.6 | 973 | 2688.1 | 1021 | **0.83** | 0.29 | 0.3442491 | 0.2477319 | **0.67** | 0.066 |
| orf19.4043 | *ASK10* | 2385.2 | 3386.2 | 2912.3 | 597 | 2424.8 | 1396 | **0.83** | 0.546 | 0.2594139 | 0.2848374 | **0.69** | 0.102 |
| orf19.4133 |  | 674.4 | 969.8 | 766.8 | 281 | 638.1 | 143 | **0.83** | 0.344 | 0.4975035 | 0.4229469 | **0.65** | 0.050 |
| orf19.4253 |  | 422.5 | 538.8 | 440.5 | 163 | 364.0 | 137 | **0.83** | 0.546 | 0.4045742 | 0.5069235 | **0.58** | 0.033 |
| orf19.4263 |  | 475.7 | 639.6 | 498.2 | 289 | 412.2 | 237 | **0.83** | 0.851 | 0.8027725 | 0.67826 | **0.59** | 0.029 |
| orf19.4293 |  | 1388.6 | 2050.8 | 1696.3 | 414 | 1401.1 | 673 | **0.83** | 0.228 | 0.2703398 | 0.0665845 | **0.60** | 0.041 |
| orf19.6671 | *LAP5* | 766.4 | 1047.7 | 870.1 | 365 | 725.2 | 394 | **0.83** | 0.291 | 0.4794051 | 0.2352198 | **0.62** | 0.074 |
| orf19.971 | *SKN7* | 726.0 | 960.6 | 854.4 | 511 | 712.5 | 510 | **0.83** | 0.475 | 0.6318694 | 0.4659096 | **0.66** | 0.074 |
| orf19.1282 | *CKS1* | 545.5 | 753.0 | 606.6 | 173 | 512.4 | 164 | **0.84** | 0.241 | 0.3551362 | 0.2256766 | **0.58** | 0.032 |
| orf19.3901 |  | 1430.4 | 2457.1 | 1772.9 | 1424 | 1489.1 | 1114 | **0.84** | 0.196 | 0.8197853 | 0.203347 | **0.62** | 0.076 |
| orf19.3936 |  | 2100.0 | 3095.5 | 2679.2 | 2309 | 2241.7 | 2627 | **0.84** | 0.306 | 0.4962811 | 0.0865377 | **0.63** | 0.041 |
| orf19.3990 | *RPC17* | 979.9 | 1507.4 | 1209.0 | 1074 | 1011.2 | 900 | **0.84** | 0.403 | 0.8547931 | 0.4730231 | **0.66** | 0.103 |
| orf19.3995 | *RIM13* | 497.9 | 662.7 | 554.3 | 237 | 467.6 | 291 | **0.84** | 0.382 | 0.4479329 | 0.3297639 | **0.59** | 0.051 |
| orf19.4024 | *RIB5* | 3538.4 | 5242.7 | 4429.1 | 2352 | 3732.0 | 2088 | **0.84** | 0.376 | 0.6218789 | 0.3511628 | **0.62** | 0.054 |
| orf19.4269 |  | 665.2 | 930.9 | 748.5 | 431 | 627.4 | 314 | **0.84** | 0.467 | 0.7686225 | 0.6105081 | **0.63** | 0.035 |
| orf19.4330 |  | 265.7 | 324.5 | 239.8 | 143 | 202.5 | 64 | **0.84** | 1.522 | 0.9661845 | 0.9688748 | **0.64** | 0.040 |
| orf19.4332 |  | 451.2 | 616.5 | 471.9 | 167 | 397.3 | 111 | **0.84** | 0.407 | 0.4621588 | 0.4243012 | **0.61** | 0.036 |
| orf19.5681 |  | 407.2 | 544.0 | 417.9 | 198 | 349.8 | 122 | **0.84** | 1.484 | 0.8762006 | 0.8645062 | **0.49** | 0.023 |
| orf19.1110 | *THI80* | 1359.3 | 2083.4 | 1578.5 | 1465 | 1334.1 | 1109 | **0.85** | 0.727 | 0.9162654 | 0.7588647 | **0.64** | 0.035 |
| orf19.1111 | *TAF9* | 669.5 | 932.5 | 792.2 | 303 | 674.6 | 308 | **0.85** | 0.387 | 0.4820985 | 0.3811505 | **0.64** | 0.033 |
| orf19.1944 | *GPR1* | 641.9 | 948.3 | 723.0 | 214 | 612.6 | 112 | **0.85** | 0.18 | 0.338845 | 0.1787692 | **0.61** | 0.050 |
| orf19.2642 | *CAM1* | 320.5 | 428.1 | 309.0 | 175 | 261.4 | 90 | **0.85** | 1.568 | 0.9711226 | 0.9713413 | **0.60** | 0.026 |
| orf19.3202 |  | 439.1 | 598.6 | 509.9 | 356 | 431.2 | 243 | **0.85** | 1.676 | 0.9940324 | 0.9929446 | **0.60** | 0.025 |
| orf19.3925^b^ |  | 189.7 | 221.4 | 150.4 | 177 | 140.8 | 38 | **0.85** | 2.569 | 0.5639059 | 0.5898505 | **0.59** | 0.035 |
| orf19.4019 |  | 347.3 | 488.1 | 364.7 | 232 | 310.2 | 95 | **0.85** | 2.18 | 0.9492438 | 0.9599931 | **0.67** | 0.106 |
| orf19.4029 | *SQT1* | 3197.4 | 4834.9 | 4034.5 | 668 | 3422.4 | 1677 | **0.85** | 0.318 | 0.2780798 | 0.1995509 | **0.58** | 0.013 |
| orf19.4245 |  | 329.1 | 426.3 | 333.9 | 183 | 285.3 | 110 | **0.85** | 1.393 | 0.965561 | 0.960647 | **0.68** | 0.053 |
| orf19.4261 | *TIF5* | 1801.6 | 2827.8 | 2182.0 | 295 | 1859.5 | 699 | **0.85** | 0.265 | 0.247721 | 0.1492363 | **0.60** | 0.019 |
| orf19.4290 | *TRR1* | 1329.6 | 1945.5 | 1614.5 | 482 | 1375.4 | 634 | **0.85** | 0.371 | 0.4061179 | 0.2984405 | **0.59** | 0.043 |
| orf19.4299 | *MSW1* | 398.4 | 553.8 | 420.7 | 178 | 356.8 | 62 | **0.85** | 1.119 | 0.858162 | 0.8700158 | **0.60** | 0.055 |
| orf19.4328 | *CCC2* | 1007.4 | 1459.3 | 1212.1 | 659 | 1028.0 | 749 | **0.85** | 0.239 | 0.5227181 | 0.1447945 | **0.56** | 0.030 |
| orf19.927 |  | 719.6 | 1130.6 | 808.0 | 227 | 684.2 | 145 | **0.85** | 0.069 | 0.4654136 | 0.0773358 | **0.62** | 0.013 |
| orf19.1109 |  | 634.4 | 948.7 | 721.6 | 173 | 623.1 | 87 | **0.86** | 0.352 | 0.4945029 | 0.6045832 | **0.57** | 0.035 |
| orf19.1281 |  | 724.2 | 1054.4 | 931.4 | 706 | 800.3 | 567 | **0.86** | 0.399 | 0.8566927 | 0.6843119 | **0.60** | 0.045 |
| orf19.2641 | *ARP1* | 488.5 | 701.0 | 496.7 | 130 | 428.0 | 170 | **0.86** | 0.292 | 0.3781664 | 0.2031118 | **0.62** | 0.027 |
| orf19.3161 |  | 1261.6 | 1862.1 | 1559.7 | 819 | 1338.0 | 935 | **0.86** | 0.285 | 0.5508137 | 0.2261874 | **0.58** | 0.063 |
| orf19.3206 | *CCT7* | 3265.9 | 5880.6 | 4039.0 | 3417 | 3492.4 | 3325 | **0.86** | 0.267 | 0.7222258 | 0.1120508 | **0.60** | 0.042 |
| orf19.3220 |  | 324.4 | 414.0 | 317.1 | 167 | 272.8 | 37 | **0.86** | 0.701 | 0.913924 | 0.9200556 | **0.65** | 0.033 |
| orf19.4217**^b^** |  | 244.2 | 295.7 | 219.6 | 181 | 188.8 | 44 | **0.86** | 6.201 | 0.7205074 | 0.7512668 | **0.63** | 0.160 |
| orf19.4270 | *MNT2* | 462.4 | 674.0 | 486.7 | 249 | 417.5 | 177 | **0.86** | 1.64 | 0.8354044 | 0.8307568 | **0.58** | 0.020 |
| orf19.4340.1 |  | 349.3 | 448.9 | 347.5 | 152 | 298.3 | 53 | **0.86** | 0.623 | 0.746179 | 0.7837211 | **0.61** | 0.027 |
| orf19.3910 | *RNY1* | 418.7 | 587.5 | 438.4 | 182 | 381.1 | 152 | **0.87** | 0.588 | 0.6698858 | 0.5980742 | **0.65** | 0.066 |
| orf19.3920 | *MPD1* | 596.2 | 926.7 | 659.6 | 167 | 571.6 | 58 | **0.87** | 0.201 | 0.3037448 | 0.2324919 | **0.64** | 0.033 |
| orf19.3947 | *SPT4* | 983.5 | 1462.9 | 1237.7 | 684 | 1079.6 | 722 | **0.87** | 0.294 | 0.6409846 | 0.2299095 | **0.48** | 0.029 |
| orf19.3967 | *PFK1* | 5393.8 | 8097.0 | 7058.8 | 3524 | 6159.3 | 4581 | **0.87** | 0.469 | 0.5117176 | 0.3069664 | **0.61** | 0.027 |
| orf19.4001 | *MSS2* | 456.3 | 618.5 | 515.4 | 449 | 446.4 | 409 | **0.87** | 1.74 | 0.993933 | 0.9902693 | **0.58** | 0.024 |
| orf19.4017**^b^** |  | 216.6 | 261.7 | 191.1 | 177 | 175.2 | 42 | **0.87** | 4.402 | 0.3174126 | 0.3382637 | **0.64** | 0.059 |
| orf19.4033 | *PRP22* | 311.7 | 407.7 | 301.4 | 175 | 263.4 | 62 | **0.87** | 1.609 | 0.9264672 | 0.9376214 | **0.63** | 0.087 |
| orf19.4275 | *RAD9* | 194.4 | 233.3 | 156.1 | 163 | 942.0 | 34 | **0.87** | 3.313 | 0.3183856 | 0.3458557 | **0.63** | 0.027 |
| orf19.4309 | *GRP3* | 1642.5 | 2563.9 | 2054.5 | 555 | 1784.2 | 708 | **0.87** | 0.326 | 0.4293759 | 0.3185885 | **0.56** | 0.006 |
| orf19.4334 | *PGA58* | 717.6 | 1138.3 | 863.7 | 447 | 748.9 | 303 | **0.87** | 0.625 | 0.8210418 | 0.7278743 | **0.59** | 0.021 |
| orf19.6673 | *HEX1* | 590.2 | 798.5 | 663.2 | 359 | 573.8 | 446 | **0.87** | 0.512 | 0.6229669 | 0.4242222 | **0.62** | 0.047 |
| orf19.3928 | *AZF2* | 482.5 | 703.0 | 514.9 | 183 | 453.9 | 129 | **0.88** | 0.317 | 0.6123386 | 0.4869997 | **0.59** | 0.044 |
| orf19.3945 |  | 679.8 | 958.2 | 813.7 | 454 | 716.7 | 534 | **0.88** | 0.542 | 0.7118354 | 0.5318297 | **0.60** | 0.049 |
| orf19.3956 |  | 405.6 | 570.6 | 427.4 | 212 | 375.3 | 137 | **0.88** | 0.585 | 0.771914 | 0.7327943 | **0.61** | 0.024 |
| orf19.3977 | *GIR1* | 588.4 | 841.2 | 642.4 | 454 | 562.2 | 377 | **0.88** | 1.806 | 0.954416 | 0.9292827 | **0.63** | 0.028 |
| orf19.4135 | *PRC2* | 1723.9 | 2733.7 | 2010.7 | 805 | 1759.4 | 622 | **0.88** | 0.216 | 0.6244605 | 0.2891632 | **0.60** | 0.097 |
| orf19.4234 |  | 2320.1 | 3769.0 | 2915.1 | 2283 | 2555.5 | 1785 | **0.88** | 0.271 | 0.8943685 | 0.5846765 | **0.54** | 0.064 |
| orf19.6656 | *DUR8* | 753.7 | 1141.4 | 871.8 | 397 | 766.8 | 276 | **0.88** | 0.493 | 0.8335715 | 0.727142 | **0.62** | 0.051 |
| orf19.925 |  | 543.8 | 827.6 | 575.6 | 417 | 505.5 | 312 | **0.88** | 1.024 | 0.996713 | 0.9941067 | **0.70** | 0.079 |
| orf19.946 | *MET14* | 1382.3 | 2312.0 | 1587.3 | 634 | 1398.5 | 384 | **0.88** | 0.244 | 0.6658269 | 0.3829969 | **0.58** | 0.025 |
| orf19.1119 | *MTR10* | 415.4 | 614.1 | 432.2 | 175 | 385.9 | 54 | **0.89** | 0.856 | 0.8611437 | 0.8804649 | **0.64** | 0.019 |
| orf19.1286**^b^** |  | 214.2 | 278.3 | 186.1 | 185 | 174.6 | 45 | **0.89** | 5.284 | 0.547876 | 0.5721267 | **0.61** | 0.036 |
| orf19.3906 |  | 483.7 | 737.0 | 538.7 | 196 | 478.9 | 87 | **0.89** | 0.615 | 0.776892 | 0.8271502 | **0.63** | 0.052 |
| orf19.4007 |  | 686.9 | 1055.3 | 821.8 | 532 | 734.2 | 418 | **0.89** | 0.389 | 0.7678175 | 0.5887082 | **0.57** | 0.038 |
| orf19.4147 | *GLR1* | 2832.4 | 4562.1 | 3644.1 | 2185 | 3241.8 | 2268 | **0.89** | 0.271 | 0.6597927 | 0.2776496 | **0.64** | 0.072 |
| orf19.4149.1**^b^** |  | 197.7 | 241.9 | 162.0 | 168 | 153.7 | 22 | **0.89** | 3.762 | 0.5472083 | 0.5622685 | **0.69** | 0.044 |
| orf19.5691 | *CDC11* | 654.3 | 991.7 | 735.9 | 494 | 655.4 | 362 | **0.89** | 0.752 | 0.9674338 | 0.9381946 | **0.64** | 0.096 |
| orf19.6668 | *CUE1* | 339.6 | 472.5 | 362.1 | 328 | 331.3 | 246 | **0.89** | 1.719 | 0.5684491 | 0.5288879 | **0.64** | 0.082 |
| orf19.961.2 |  | 1780.7 | 2865.5 | 2280.5 | 907 | 2030.2 | 936 | **0.89** | 0.3 | 0.6668439 | 0.3777562 | **0.56** | 0.021 |
| orf19.1926 | *SEF2* | 595.7 | 877.2 | 675.5 | 402 | 609.5 | 398 | **0.9** | 0.453 | 0.7961345 | 0.6414924 | **0.61** | 0.029 |
| orf19.2643 | *RPO26* | 2663.0 | 4497.9 | 3483.0 | 1081 | 3147.1 | 1061 | **0.9** | 0.221 | 0.604791 | 0.3406533 | **0.58** | 0.038 |
| orf19.3209 | *FGR42* | 433.7 | 626.5 | 490.5 | 471 | 442.6 | 340 | **0.9** | 38.11 | 0.5952767 | 0.5781505 | **0.73** | 0.026 |
| orf19.3899 |  | 364.6 | 513.2 | 364.6 | 144 | 327.1 | 29 | **0.9** | 0.528 | 0.8415751 | 0.8652955 | **0.63** | 0.098 |
| orf19.3902 |  | 277.8 | 379.6 | 270.7 | 177 | 243.0 | 43 | **0.9** | 34.99 | 0.5001461 | 0.5103835 | **0.62** | 0.033 |
| orf19.4012 | *PCL5* | 777.4 | 1211.7 | 933.9 | 312 | 840.5 | 387 | **0.9** | 0.239 | 0.574977 | 0.3271243 | **0.63** | 0.025 |
| orf19.4018 | *RSM7* | 627.2 | 989.7 | 691.4 | 399 | 625.5 | 323 | **0.9** | 0.413 | 0.8979009 | 0.7641563 | **0.59** | 0.034 |
| orf19.4025 | *PRE1* | 3547.0 | 5918.2 | 4573.7 | 1453 | 4137.3 | 1925 | **0.9** | 0.211 | 0.5525114 | 0.1578677 | **0.64** | 0.092 |
| orf19.4241 |  | 678.5 | 1102.9 | 780.0 | 222 | 701.7 | 48 | **0.9** | 0.238 | 0.7052767 | 0.6428719 | **0.49** | 0.064 |
| orf19.575 | *HYR5* | 968.5 | 1374.3 | 1156.9 | 575 | 1042.4 | 902 | **0.9** | 0.409 | 0.5318211 | 0.3117983 | **0.49** | 0.027 |
| orf19.938 |  | 215.3 | 261.0 | 179.9 | 148 | 236.0 | 39 | **0.9** | 15.27 | 0.3651197 | 0.3925818 | **0.59** | 0.023 |
| orf19.954 | *XDJ1* | 417.4 | 595.3 | 434.1 | 208 | 392.1 | 84 | **0.9** | 0.663 | 0.9263537 | 0.9378285 | **0.62** | 0.070 |
| orf19.1939^b^ |  | 231.5 | 302.6 | 203.6 | 167 | 185.6 | 52 | **0.91** | 130.2 | 0.3271781 | 0.3590137 | **0.61** | 0.054 |
| orf19.1943 |  | 366.4 | 515.3 | 372.8 | 156 | 338.3 | 68 | **0.91** | 1.029 | 0.9455817 | 0.9564823 | **0.52** | 0.050 |
| orf19.1950 |  | 1028.0 | 1650.3 | 1248.2 | 1115 | 1131.8 | 991 | **0.91** | 1.935 | 0.8179238 | 0.614747 | **0.72** | 0.067 |
| orf19.1978 | *GIT2* | 346.0 | 489.9 | 337.6 | 126 | 307.9 | 91 | **0.91** | 0.726 | 0.7385208 | 0.7940995 | **0.64** | 0.032 |
| orf19.2650.1 |  | 507.3 | 747.9 | 562.1 | 400 | 510.5 | 384 | **0.91** | 3.793 | 0.8657889 | 0.8171741 | **0.59** | 0.053 |
| orf19.3903 |  | 363.3 | 546.5 | 362.9 | 198 | 330.4 | 67 | **0.91** | 0.669 | 0.9881552 | 0.9876138 | **0.61** | 0.053 |
| orf19.4046 |  | 391.4 | 580.1 | 434.1 | 270 | 396.4 | 105 | **0.91** | 1.101 | 0.9037448 | 0.8979159 | **0.60** | 0.098 |
| orf19.6672 | *MDJ1* | 4818.1 | 8987.1 | 6130.5 | 6172 | 5590.2 | 5926 | **0.91** | 0.336 | 0.8325215 | 0.2140752 | **0.59** | 0.038 |
| orf19.1955 | *SHR5* | 317.4 | 428.2 | 312.4 | 171 | 286.0 | 64 | **0.92** | 1.038 | 0.8413928 | 0.840282 | **0.65** | 0.038 |
| orf19.2637 | *PER1* | 747.9 | 1119.3 | 927.4 | 988 | 855.8 | 1015 | **0.92** | 6.75 | 0.6348187 | 0.5333666 | **0.59** | 0.026 |
| orf19.2646 | *HAP4/HAP1* | 636.4 | 1031.6 | 757.1 | 507 | 694.1 | 418 | **0.92** | 0.533 | 0.7988293 | 0.6541789 | **0.68** | 0.073 |
| orf19.3210^b^ |  | 193.6 | 251.1 | 157.1 | 183 | 159.5 | 26 | **0.92** | 2.558 | 0.5377309 | 0.5543322 | **0.54** | 0.025 |
| orf19.3904b |  | 214.6 | 285.3 | 185.8 | 176 | 180.7 | 43 | **0.92** | 4.894 | 0.2947509 | 0.3169955 | **0.63** | 0.027 |
| orf19.3921 | *FSH3* | 648.6 | 1082.9 | 761.5 | 248 | 703.9 | 103 | **0.92** | 0.528 | 0.8259038 | 0.8564906 | **0.65** | 0.084 |
| orf19.4045 | *EST1* | 761.8 | 1194.1 | 879.4 | 681 | 809.6 | 617 | **0.92** | 3.559 | 0.7987052 | 0.6663976 | **0.66** | 0.083 |
| orf19.4287 |  | 522.4 | 832.1 | 580.6 | 309 | 536.9 | 235 | **0.92** | 0.577 | 0.9764897 | 0.9624456 | **0.57** | 0.036 |
| orf19.4306 |  | 748.2 | 1222.2 | 844.2 | 270 | 774.5 | 255 | **0.92** | 0.344 | 0.6530071 | 0.5136985 | **0.61** | 0.026 |
| orf19.581 | *NRD1* | 571.0 | 900.6 | 621.9 | 228 | 574.9 | 75 | **0.92** | 0.32 | 0.8114459 | 0.8198469 | **0.67** | 0.041 |
| orf19.1121 |  | 1982.8 | 3315.9 | 2510.8 | 1943 | 2330.4 | 2193 | **0.93** | 0.528 | 0.883551 | 0.6576533 | **0.63** | 0.047 |
| orf19.3926 | *RNY1* | 417.7 | 632.0 | 478.1 | 337 | 446.0 | 181 | **0.93** | 1.691 | 0.8157009 | 0.8100083 | **0.56** | 0.029 |
| orf19.3952 | *SRP101* | 555.7 | 810.9 | 612.5 | 173 | 567.9 | 238 | **0.93** | 0.386 | 0.6048472 | 0.6217548 | **0.58** | 0.029 |
| orf19.4229 | *DDP1* | 842.0 | 1282.3 | 1017.6 | 552 | 941.7 | 640 | **0.93** | 0.295 | 0.700433 | 0.4029203 | **0.61** | 0.072 |
| orf19.4243^b^ | *YKL090* | 231.1 | 316.2 | 200.7 | 173 | 191.7 | 46 | **0.93** | 3.762 | 0.412483 | 0.422552 | **0.39** | 0.027 |
| orf19.4248 |  | 2930.3 | 5152.7 | 3894.6 | 2390 | 3608.5 | 2251 | **0.93** | 0.203 | 0.7682182 | 0.2650192 | **0.58** | 0.074 |
| orf19.4305 |  | 259.9 | 334.0 | 241.8 | 171 | 224.6 | 98 | **0.93** | 3.469 | 0.6619762 | 0.6791614 | **0.53** | 0.031 |
| orf19.4310b |  | 181.4 | 227.4 | 139.7 | 168 | 145.4 | 45 | **0.93** | 2.589 | 0.5436185 | 0.5745209 | **0.48** | 0.023 |
| orf19.4340 |  | 258.1 | 360.9 | 235.6 | 197 | 227.2 | 84 | **0.93** | 2.451 | 0.4715173 | 0.4686619 | **0.52** | 0.025 |
| orf19.570b | *HYR9* | 229.5 | 311.9 | 203.8 | 172 | 194.4 | 46 | **0.93** | 7.683 | 0.3687502 | 0.3851586 | **0.66** | 0.037 |
| orf19.944 | *IFG3* | 1202.9 | 2004.4 | 1482.0 | 849 | 1378.6 | 568 | **0.93** | 0.41 | 0.984579 | 0.9672513 | **0.59** | 0.026 |
| orf19.955 |  | 602.6 | 897.2 | 670.3 | 186 | 623.9 | 234 | **0.93** | 0.383 | 0.6178011 | 0.6484656 | **0.58** | 0.024 |
| orf19.3208 | *DAL52* | 272.9 | 379.0 | 258.6 | 196 | 247.0 | 117 | **0.94** | 3.601 | 0.5039444 | 0.483467 | **0.62** | 0.035 |
| orf19.3930 | *YUH2* | 775.1 | 1255.3 | 918.9 | 325 | 862.7 | 298 | **0.94** | 0.416 | 0.7943206 | 0.7612198 | **0.58** | 0.026 |
| orf19.4006 | *PAN5* | 309.0 | 439.3 | 309.1 | 186 | 290.9 | 52 | **0.94** | 2.747 | 0.6336804 | 0.6558237 | **0.58** | 0.034 |
| orf19.4054 | *CTA24* | 9090.8 | 18020.9 | 11659.2 | 8805 | 10969.4 | 10128 | **0.94** | 0.415 | 0.7801715 | 0.4438239 | **0.88** | 0.082 |
| orf19.4266 | *SPR28* | 315.9 | 447.1 | 312.3 | 183 | 297.2 | 81 | **0.94** | 7.719 | 0.4932406 | 0.4814061 | **0.60** | 0.024 |
| orf19.1980 | *GIT4* | 249.5 | 335.9 | 225.0 | 156 | 212.8 | 76 | **0.95** | 14.5 | 0.5235171 | 0.5566724 | **0.62** | 0.048 |
| orf19.2657 |  | 478.1 | 763.4 | 502.4 | 226 | 475.6 | 196 | **0.95** | 0.725 | 0.9342424 | 0.9088714 | **1.01** | 0.114 |
| orf19.3968^b^ |  | 191.0 | 269.1 | 154.2 | 194 | 166.8 | 42 | **0.95** | 2.3 | 0.5297787 | 0.5524604 | **0.63** | 0.034 |
| orf19.936 |  | 345.4 | 537.4 | 357.5 | 182 | 341.4 | 142 | **0.95** | 1.639 | 0.8607906 | 0.8802952 | **0.51** | 0.065 |
| orf19.1122 |  | 283.8 | 398.6 | 263.2 | 177 | 252.4 | 66 | **0.96** | 14.73 | 0.5263211 | 0.5393645 | **0.73** | 0.113 |
| orf19.2653 |  | 236.2 | 328.4 | 209.9 | 171 | 203.6 | 55 | **0.96** | 19.4 | 0.3544235 | 0.389244 | **0.60** | 0.037 |
| orf19.3176 | *RIM21* | 522.2 | 833.6 | 587.5 | 213 | 565.5 | 144 | **0.96** | 0.498 | 0.9912381 | 0.991049 | **0.54** | 0.049 |
| orf19.3200b | *MATa2* | 197.6 | 258.7 | 161.0 | 152 | 164.5 | 72 | **0.96** | 5.128 | 0.3039216 | 0.3488264 | **0.05** | 0.010 |
| orf19.3942.1^b^ | *VPS25* | 229.8 | 312.6 | 198.4 | 170 | 194.1 | 53 | **0.96** | 5.017 | 0.4001515 | 0.4192677 | **0.67** | 0.046 |
| orf19.3963 |  | 252.1 | 329.2 | 228.2 | 158 | 219.3 | 49 | **0.96** | 1.729 | 0.7441192 | 0.7253383 | **0.61** | 0.035 |
| orf19.3971 |  | 296.3 | 449.9 | 276.4 | 186 | 265.6 | 97 | **0.96** | 1.794 | 0.719519 | 0.7157164 | **0.62** | 0.030 |
| orf19.3973 |  | 1193.7 | 2016.7 | 1571.6 | 906 | 1511.7 | 945 | **0.96** | 0.19 | 0.8491485 | 0.4793759 | **0.62** | 0.028 |
| orf19.4028 | *RER2* | 458.3 | 748.5 | 499.9 | 169 | 481.7 | 39 | **0.96** | 0.601 | 0.9077743 | 0.9197132 | **0.65** | 0.080 |
| orf19.4231 | *PTH2* | 254.3 | 339.1 | 229.4 | 156 | 219.9 | 55 | **0.96** | 3.359 | 0.5568769 | 0.6235697 | **0.58** | 0.024 |
| orf19.4244^b^ | *YIL130* | 205.2 | 263.7 | 168.7 | 154 | 168.7 | 46 | **0.96** | 47.31 | 0.295154 | 0.3297585 | **0.66** | 0.040 |
| orf19.4320b |  | 210.4 | 277.2 | 176.3 | 177 | 175.4 | 35 | **0.96** | 7.03 | 0.5254415 | 0.5454884 | **0.68** | 0.058 |
| orf19.4349 | *KRE12* | 2011.4 | 3838.2 | 2309.6 | 879 | 2224.2 | 1184 | **0.96** | 0.239 | 0.7527094 | 0.3142762 | **0.51** | 0.025 |
| orf19.5682 | *SRP1* | 928.3 | 1481.1 | 1072.5 | 165 | 1030.5 | 305 | **0.96** | 0.392 | 0.6344666 | 0.7180839 | **0.61** | 0.111 |
| orf19.584 | *CCP2* | 240.7 | 332.7 | 215.7 | 167 | 212.9 | 89 | **0.96** | 3.277 | 0.4604887 | 0.4744873 | **0.66** | 0.094 |
| orf19.6681 |  | 370.1 | 547.2 | 380.9 | 184 | 364.6 | 92 | **0.96** | 0.846 | 0.8512179 | 0.8662374 | **0.60** | 0.069 |
| orf19.921 |  | 311.7 | 450.9 | 318.6 | 241 | 310.7 | 166 | **0.96** | 3.328 | 0.4792303 | 0.4505793 | **0.60** | 0.029 |
| orf19.929b |  | 198.3 | 262.3 | 159.8 | 157 | 163.1 | 48 | **0.96** | 5.628 | 0.2902697 | 0.3241224 | **0.61** | 0.033 |
| orf19.1112 | *BUD7* | 472.4 | 727.3 | 497.0 | 224 | 484.2 | 229 | **0.97** | 0.582 | 0.9045648 | 0.8982089 | **0.60** | 0.028 |
| orf19.1113 |  | 552.7 | 921.1 | 629.5 | 479 | 612.1 | 411 | **0.97** | 2.244 | 0.8377822 | 0.7626713 | **0.57** | 0.030 |
| orf19.1120 |  | 364.5 | 593.6 | 376.2 | 170 | 366.0 | 67 | **0.97** | 0.932 | 0.8913219 | 0.9095218 | **0.61** | 0.057 |
| orf19.1124 | *DPH52* | 1160.2 | 2015.2 | 1413.4 | 932 | 1368.3 | 974 | **0.97** | 0.272 | 0.9350722 | 0.7659806 | **0.62** | 0.056 |
| orf19.2640 | *FUR1* | 1470.8 | 2716.2 | 1867.9 | 1199 | 1811.1 | 1245 | **0.97** | 0.307 | 0.8428784 | 0.5048154 | **0.59** | 0.053 |
| orf19.2645 |  | 293.4 | 427.4 | 301.8 | 249 | 293.5 | 92 | **0.97** | 12.16 | 0.6179355 | 0.6494813 | **0.57** | 0.040 |
| orf19.3203 | *RCY1* | 285.3 | 417.2 | 270.1 | 185 | 261.0 | 47 | **0.97** | 3.059 | 0.5291332 | 0.5455875 | **0.63** | 0.028 |
| orf19.3970 |  | 469.9 | 719.4 | 520.9 | 218 | 504.2 | 218 | **0.97** | 0.615 | 0.9725669 | 0.9716932 | **0.60** | 0.033 |
| orf19.4273 | *SLS1* | 508.6 | 803.3 | 571.9 | 468 | 552.7 | 456 | **0.97** | 34.31 | 0.5758747 | 0.5223998 | **0.61** | 0.028 |
| orf19.1124.2 |  | 238.5 | 336.3 | 213.3 | 157 | 213.2 | 73 | **0.98** | 3.767 | 0.4416671 | 0.4427766 |  |  |
| orf19.1932b | *FRE5* | 213.5 | 280.7 | 181.5 | 159 | 183.8 | 61 | **0.98** | 8.229 | 0.286677 | 0.3249624 | **0.67** | 0.077 |
| orf19.1938 |  | 328.1 | 507.1 | 324.5 | 171 | 317.7 | 96 | **0.98** | 1.433 | 0.7575943 | 0.7866539 | **0.64** | 0.055 |
| orf19.1970 | *CVB1* | 472.6 | 705.5 | 539.2 | 354 | 525.8 | 452 | **0.98** | 0.481 | 0.8979666 | 0.8308293 | **0.61** | 0.038 |
| orf19.2639.1 | *MRPL20* | 737.8 | 1244.2 | 827.8 | 571 | 811.3 | 444 | **0.98** | 1.509 | 0.8755933 | 0.820505 | **0.56** | 0.051 |
| orf19.3924b |  | 195.7 | 259.1 | 158.7 | 157 | 167.1 | 45 | **0.98** | 4.961 | 0.284934 | 0.317713 | **0.64** | 0.040 |
| orf19.4026 | *HIS1* | 4396.1 | 8473.1 | 5438.1 | 603 | 5336.2 | 2071 | **0.98** | 0.426 | 0.66162 | 0.7048112 | **0.62** | 0.055 |
| orf19.4323 |  | 333.6 | 491.6 | 337.6 | 176 | 332.0 | 81 | **0.98** | 0.853 | 0.8379563 | 0.8312532 | **0.61** | 0.060 |
| orf19.1952 |  | 265.7 | 392.1 | 247.1 | 181 | 244.4 | 60 | **0.99** | 3.268 | 0.7011586 | 0.7412818 |  |  |
| orf19.1969^b^ | *CCW14* | 213.5 | 295.0 | 180.4 | 167 | 185.7 | 68 | **0.99** | 7.526 | 0.2841439 | 0.3264844 | **0.67** | 0.051 |
| orf19.1975 | *DIB1* | 333.0 | 526.2 | 356.1 | 302 | 362.5 | 201 | **0.99** | 2.243 | 0.4578811 | 0.4185002 | **0.68** | 0.099 |
| orf19.3173 | *SSP120* | 1315.3 | 2308.2 | 1717.1 | 1482 | 1696.0 | 1340 | **0.99** | 0.29 | 0.990725 | 0.9662089 | **0.62** | 0.034 |
| orf19.3965 |  | 267.4 | 401.2 | 252.1 | 180 | 250.6 | 31 | **0.99** | 2.978 | 0.6806766 | 0.7014656 | **0.62** | 0.046 |
| orf19.4005 | *YDR196* | 539.6 | 881.8 | 633.4 | 548 | 627.7 | 521 | **0.99** | 3.907 | 0.7129059 | 0.5867718 | **0.63** | 0.058 |
| orf19.4151 | *SPO1* | 264.8 | 411.5 | 249.5 | 211 | 249.9 | 143 | **0.99** | 6.827 | 0.4251124 | 0.440143 | **0.63** | 0.055 |
| orf19.4250^b^ |  | 231.1 | 310.2 | 199.6 | 155 | 197.7 | 30 | **0.99** | 4.091 | 0.6571112 | 0.6866094 | **0.69** | 0.073 |
| orf19.4264 |  | 254.6 | 378.7 | 233.2 | 174 | 240.8 | 99 | **0.99** | 1.851 | 0.4718313 | 0.4604358 | **0.63** | 0.031 |
| orf19.4316 |  | 617.0 | 1054.3 | 693.9 | 301 | 684.4 | 200 | **0.99** | 0.457 | 0.8763111 | 0.8082472 | **0.56** | 0.027 |
| orf19.578 | *MSB3* | 368.5 | 548.0 | 383.1 | 212 | 378.7 | 195 | **0.99** | 1.081 | 0.8305185 | 0.8003896 | **0.62** | 0.026 |
| orf19.1280 | *SUI1* | 5899.3 | 10442.5 | 8481.5 | 7203 | 8466.1 | 9430 | **1** | 0.274 | 0.7823127 | 0.289133 | **0.63** | 0.036 |
| orf19.1976 | *TRX1* | 448.0 | 732.9 | 510.4 | 389 | 509.0 | 329 | **1** | 7.033 | 0.6321294 | 0.5618638 | **0.57** | 0.055 |
| orf19.3905 |  | 280.8 | 412.1 | 267.2 | 181 | 267.4 | 76 | **1** | 2.674 | 0.7290505 | 0.7802388 | **0.52** | 0.039 |
| orf19.3916 |  | 677.8 | 1171.7 | 823.3 | 624 | 827.1 | 491 | **1** | 0.687 | 0.7951513 | 0.6066391 | **0.63** | 0.026 |
| orf19.3986 | *PPR1* | 292.9 | 424.0 | 278.7 | 153 | 277.4 | 110 | **1** | 2.111 | 0.6838898 | 0.6845807 | **0.62** | 0.023 |
| orf19.4228 |  | 849.1 | 1544.3 | 1032.5 | 536 | 1032.5 | 427 | **1** | 0.475 | 0.8882825 | 0.8173072 | **0.59** | 0.033 |
| orf19.6667 | *SAP30* | 994.5 | 1711.2 | 1303.7 | 836 | 1308.3 | 856 | **1** | 0.436 | 0.9928126 | 0.9846464 | **0.61** | 0.070 |
| orf19.948^b^ |  | 205.8 | 280.7 | 174.8 | 175 | 183.8 | 34 | **1** | 5.102 | 0.5180102 | 0.5356138 | **0.58** | 0.019 |
| orf19.1981 | *IMP2* | 415.7 | 698.0 | 481.5 | 412 | 488.7 | 314 | **1.01** | 1.747 | 0.6346338 | 0.5441117 | **0.94** | 0.218 |
| orf19.3171 | *ACH1* | 3227.4 | 6400.6 | 3847.8 | 1494 | 3889.6 | 1504 | **1.01** | 0.303 | 0.9028311 | 0.8159555 | **0.56** | 0.025 |
| orf19.3184 | *SFT2b* | 1730.4 | 2920.6 | 2239.8 | 1158 | 2258.5 | 1746 | **1.01** | 0.323 | 0.7413605 | 0.4808806 | **0.59** | 0.024 |
| orf19.4216 | *HSP122* | 4065.6 | 8170.7 | 5181.6 | 5541 | 5240.2 | 5631 | **1.01** | 5.865 | 0.8258149 | 0.5477539 |  |  |
| orf19.4221 | *ORC4* | 565.6 | 961.4 | 621.9 | 150 | 629.5 | 154 | **1.01** | 0.472 | 0.9188911 | 0.9345946 | **0.58** | 0.061 |
| orf19.4227 |  | 481.9 | 820.2 | 549.6 | 500 | 559.5 | 455 | **1.01** | 2.331 | 0.5420303 | 0.4566839 | **0.60** | 0.017 |
| orf19.4315 |  | 359.2 | 549.5 | 363.3 | 207 | 366.8 | 106 | **1.01** | 2.313 | 0.6154428 | 0.6283195 | **0.57** | 0.024 |
| orf19.937 |  | 298.1 | 438.3 | 289.6 | 154 | 292.1 | 118 | **1.01** | 1.833 | 0.8513337 | 0.8934757 | **0.44** | 0.030 |
| orf19.3178 | *PRP9* | 366.3 | 579.0 | 367.6 | 204 | 374.5 | 173 | **1.02** | 0.976 | 0.7634873 | 0.6966098 | **0.61** | 0.051 |
| orf19.3186 |  | 273.9 | 398.7 | 268.0 | 199 | 272.4 | 73 | **1.02** | 4.191 | 0.6589354 | 0.6864747 | **0.63** | 0.062 |
| orf19.3212 | *MID1* | 788.2 | 1325.9 | 919.2 | 273 | 938.3 | 358 | **1.02** | 0.422 | 0.9941787 | 0.9930898 | **0.64** | 0.048 |
| orf19.3929 |  | 608.0 | 1022.0 | 695.2 | 477 | 710.1 | 548 | **1.02** | 0.568 | 0.9338745 | 0.8452275 | **0.56** | 0.043 |
| orf19.4044 | *MUM2* | 496.7 | 843.4 | 556.0 | 286 | 564.4 | 223 | **1.02** | 0.833 | 0.7598173 | 0.727175 | **0.65** | 0.131 |
| orf19.4153 | *ULA1* | 263.4 | 390.2 | 248.6 | 184 | 257.4 | 117 | **1.02** | 4.826 | 0.4634288 | 0.4803031 | **0.66** | 0.014 |
| orf19.6674 | *BTS1* | 434.8 | 711.3 | 470.0 | 241 | 481.7 | 218 | **1.02** | 1.291 | 0.7127706 | 0.6394177 | **0.60** | 0.067 |
| orf19.1954 | *PUS4* | 761.5 | 1287.3 | 885.9 | 367 | 910.5 | 505 | **1.03** | 0.723 | 0.9553283 | 0.9484454 | **0.62** | 0.057 |
| orf19.3187 | *HAL9e* | 355.2 | 567.2 | 356.3 | 145 | 368.0 | 120 | **1.03** | 1.54 | 0.7477351 | 0.7975441 | **0.51** | 0.023 |
| orf19.3204 |  | 382.8 | 624.7 | 407.0 | 294 | 423.7 | 274 | **1.03** | 5.117 | 0.4912741 | 0.4547608 | **0.66** | 0.037 |
| orf19.3213 |  | 408.0 | 663.8 | 420.0 | 207 | 432.4 | 173 | **1.03** | 1.954 | 0.686454 | 0.6506941 | **0.61** | 0.031 |
| orf19.3908 |  | 959.8 | 1746.3 | 1166.5 | 376 | 1199.3 | 405 | **1.03** | 0.021 | 0.9265242 | 0.1664146 | **0.53** | 0.036 |
| orf19.4144 |  | 652.4 | 1092.5 | 774.9 | 647 | 795.0 | 710 | **1.03** | 1.997 | 0.8127654 | 0.6871636 | **0.67** | 0.061 |
| orf19.4232 | *PTH1* | 818.2 | 1315.8 | 927.2 | 352 | 951.8 | 485 | **1.03** | 0.725 | 0.9958829 | 0.9962522 | **0.65** | 0.059 |
| orf19.4239^b^ |  | 203.2 | 281.8 | 164.3 | 147 | 178.2 | 89 | **1.03** | 4.564 | 0.4020473 | 0.4354441 | **0.65** | 0.037 |
| orf19.4307 | *FTI1* | 502.1 | 856.4 | 605.8 | 645 | 627.0 | 658 | **1.03** | 2.564 | 0.7323454 | 0.5828998 | **0.53** | 0.018 |
| orf19.4326 |  | 292.7 | 413.1 | 271.5 | 146 | 279.9 | 79 | **1.03** | 1.284 | 0.6096514 | 0.6475945 | **0.58** | 0.032 |
| orf19.1968 | *MAK31* | 342.9 | 535.0 | 336.0 | 185 | 349.0 | 175 | **1.04** | 6.5 | 0.6135289 | 0.5926256 | **0.61** | 0.033 |
| orf19.2638 |  | 227.8 | 342.2 | 196.4 | 158 | 208.0 | 86 | **1.04** | 5.094 | 0.3990336 | 0.4254146 | **0.59** | 0.033 |
| orf19.2652 |  | 220.1 | 323.4 | 193.7 | 166 | 209.5 | 83 | **1.04** | 9.722 | 0.3429154 | 0.3716136 | **0.50** | 0.030 |
| orf19.3894 |  | 507.7 | 797.4 | 565.7 | 230 | 590.7 | 354 | **1.04** | 0.526 | 0.9654736 | 0.9603059 | **0.58** | 0.031 |
| orf19.4251 |  | 273.8 | 417.5 | 256.3 | 170 | 266.6 | 111 | **1.04** | 3.005 | 0.5678815 | 0.6479936 | **0.69** | 0.088 |
| orf19.1105.3 |  | 247.8 | 366.4 | 237.8 | 211 | 250.1 | 66 | **1.05** | 134.5 | 0.5045917 | 0.5168987 | **0.68** | 0.094 |
| orf19.1957 | *CYC3* | 1499.3 | 2748.2 | 1975.0 | 892 | 2075.5 | 1270 | **1.05** | 0.345 | 0.9685194 | 0.9399919 | **0.64** | 0.043 |
| orf19.3922 | *SDT1* | 1233.7 | 2106.8 | 1676.2 | 1309 | 1764.0 | 2063 | **1.05** | 0.385 | 0.7458558 | 0.4018049 | **0.65** | 0.042 |
| orf19.4321 |  | 230.9 | 343.8 | 203.6 | 189 | 215.9 | 73 | **1.05** | 60.05 | 0.5057577 | 0.537701 | **0.69** | 0.052 |
| orf19.6679 | *ALD1* | 422.3 | 725.7 | 455.4 | 205 | 477.0 | 104 | **1.05** | 0.753 | 0.5932505 | 0.595142 | **0.64** | 0.121 |
| orf19.933 | *UBC13* | 2027.7 | 3797.1 | 2671.9 | 1303 | 2801.6 | 1621 | **1.05** | 0.482 | 0.9531403 | 0.9108451 |  |  |
| orf19.1106 |  | 230.6 | 344.2 | 201.3 | 158 | 214.6 | 73 | **1.06** | 33.65 | 0.3320175 | 0.3538382 | **0.60** | 0.042 |
| orf19.2650 | *MRP10* | 308.5 | 555.5 | 306.8 | 177 | 324.4 | 155 | **1.06** | 1.787 | 0.6516676 | 0.6854959 | **0.66** | 0.029 |
| orf19.3983 |  | 2029.8 | 4215.3 | 2567.7 | 1465 | 2729.9 | 1693 | **1.06** | 0.423 | 0.9842847 | 0.9672675 | **0.60** | 0.025 |
| orf19.4134 |  | 568.8 | 958.6 | 625.6 | 179 | 665.4 | 236 | **1.06** | 0.531 | 0.8158129 | 0.84156 | **0.63** | 0.057 |
| orf19.4222 | *SST2* | 293.1 | 440.1 | 282.7 | 173 | 300.1 | 124 | **1.06** | 1.534 | 0.5501237 | 0.5354158 | **0.59** | 0.037 |
| orf19.3897 |  | 268.1 | 415.5 | 251.6 | 181 | 270.2 | 119 | **1.07** | 4.388 | 0.4650872 | 0.5020796 | **0.60** | 0.048 |
| orf19.3907^b^ |  | 191.4 | 278.7 | 150.6 | 171 | 174.1 | 73 | **1.07** | 4.336 | 0.5102851 | 0.5500525 | **0.60** | 0.023 |
| orf19.4132 |  | 10177.3 | 18548.3 | 12979.4 | 2934 | 13946.6 | 11805 | **1.07** | 0.723 | 0.7930156 | 0.7776996 | **0.66** | 0.016 |
| orf19.4286 |  | 383.9 | 608.0 | 433.2 | 310 | 461.5 | 370 | **1.07** | 1.296 | 0.7076615 | 0.6128811 | **0.66** | 0.051 |
| orf19.6678 |  | 221.2 | 321.5 | 192.0 | 155 | 211.0 | 80 | **1.07** | 5.885 | 0.3586848 | 0.3801849 | **0.63** | 0.077 |
| orf19.1930 | *FRE4* | 209.0 | 314.4 | 180.8 | 172 | 207.2 | 71 | **1.08** | 4.581 | 0.2581969 | 0.2857615 | **0.59** | 0.041 |
| orf19.3172 |  | 227.5 | 341.0 | 203.4 | 180 | 222.4 | 62 | **1.08** | 38.98 | 0.4995699 | 0.5275567 | **0.66** | 0.075 |
| orf19.3988 |  | 382.5 | 632.4 | 428.6 | 297 | 463.2 | 245 | **1.08** | 1.439 | 0.5596442 | 0.4531477 | **0.65** | 0.032 |
| orf19.3998 |  | 353.6 | 608.9 | 366.5 | 176 | 396.4 | 48 | **1.08** | 1.082 | 0.6620364 | 0.7007493 | **0.64** | 0.060 |
| orf19.3935 |  | 439.0 | 757.6 | 497.4 | 286 | 542.7 | 229 | **1.09** | 1.167 | 0.7851433 | 0.7929528 | **0.72** | 0.081 |
| orf19.4014 | *CDC102* | 342.5 | 529.1 | 363.5 | 287 | 395.9 | 339 | **1.09** | 41.76 | 0.4896737 | 0.4534798 | **0.65** | 0.075 |
| orf19.4305.1 |  | 282.6 | 465.1 | 268.3 | 144 | 292.5 | 108 | **1.09** | 2.769 | 0.5298607 | 0.6246247 | **0.65** | 0.086 |
| orf19.3966 | *CRH2* | 227.2 | 348.7 | 200.9 | 164 | 224.5 | 63 | **1.1** | 16.48 | 0.2845148 | 0.3229019 | **0.64** | 0.051 |
| orf19.4246 | *YKR070W* | 1220.1 | 2278.5 | 1499.4 | 538 | 1646.7 | 1378 | **1.1** | 0.459 | 0.8212346 | 0.6972124 | **0.61** | 0.035 |
| orf19.4339 | *VSP4* | 662.8 | 1164.7 | 751.0 | 558 | 828.7 | 744 | **1.1** | 1.382 | 0.8124599 | 0.6505582 | **0.66** | 0.046 |
| orf19.935 | *AGA1* | 1525.7 | 3080.8 | 2004.8 | 1609 | 2205.7 | 1666 | **1.1** | 0.254 | 0.8330139 | 0.3856459 | **0.58** | 0.024 |
| orf19.3196 |  | 335.5 | 593.2 | 347.7 | 191 | 386.1 | 171 | **1.11** | 2.075 | 0.7182802 | 0.7992638 | **0.23** | 0.019 |
| orf19.6296 | *SNF8* | 346.8 | 613.5 | 345.9 | 192 | 387.9 | 145 | **1.12** | 1.998 | 0.4796287 | 0.5792035 | **0.60** | 0.024 |
| orf19.942b | *KRE8* | 204.8 | 308.2 | 169.6 | 153 | 198.0 | 81 | **1.12** | 16.61 | 0.2558341 | 0.3056543 | **0.58** | 0.025 |
| orf19.3168 | *RPN8* | 1654.4 | 3307.2 | 2157.8 | 2082 | 2440.1 | 2357 | **1.13** | 0.516 | 0.7403461 | 0.2422203 | **0.62** | 0.061 |
| orf19.3898 | *TLG1* | 261.4 | 413.0 | 243.5 | 181 | 278.6 | 141 | **1.13** | 7.178 | 0.4105772 | 0.4334357 | **0.66** | 0.036 |
| orf19.4284 | *PCH1* | 1000.8 | 2038.8 | 1199.4 | 1029 | 1355.3 | 1277 | **1.13** | 1.101 | 0.8400493 | 0.6189714 | **0.67** | 0.019 |
| orf19.3991 |  | 1611.9 | 3454.2 | 2071.4 | 1646 | 2366.6 | 2044 | **1.14** | 0.431 | 0.999237 | 0.9976913 | **0.63** | 0.033 |
| orf19.4143 | *FYV7* | 358.2 | 617.4 | 372.3 | 245 | 425.7 | 263 | **1.14** | 2.999 | 0.5129507 | 0.4208819 | **0.66** | 0.060 |
| orf19.4252 | *BUD32* | 461.1 | 825.7 | 497.3 | 226 | 568.6 | 231 | **1.14** | 0.79 | 0.5137321 | 0.50727 | **0.48** | 0.014 |
| orf19.4292 | *PEP12* | 526.5 | 943.1 | 614.2 | 357 | 698.5 | 435 | **1.14** | 0.907 | 0.6779778 | 0.5028332 | **0.59** | 0.039 |
| orf19.1979 | *GIT3* | 873.0 | 1629.3 | 1075.3 | 488 | 1238.4 | 832 | **1.15** | 0.47 | 0.8605136 | 0.7975167 | **0.65** | 0.057 |
| orf19.2655 | *BUB3* | 1372.0 | 2651.7 | 1873.8 | 1959 | 2145.6 | 2626 | **1.15** | 0.45 | 0.9221323 | 0.7752136 | **0.61** | 0.039 |
| orf19.3214 |  | 616.5 | 1220.3 | 723.6 | 357 | 837.5 | 308 | **1.16** | 0.625 | 0.4662853 | 0.3967278 | **0.70** | 0.045 |
| orf19.580 |  | 340.8 | 574.6 | 349.6 | 171 | 406.1 | 158 | **1.16** | 1.051 | 0.4633011 | 0.4741963 | **0.68** | 0.051 |
| orf19.2651 | *TEF4* | 11266.3 | 26674.8 | 14549.4 | 14215 | 16966.1 | 18041 | **1.17** | 0.403 | 0.9327373 | 0.6183705 | **0.62** | 0.044 |
| orf19.4011 |  | 219.6 | 348.9 | 189.9 | 155 | 225.9 | 99 | **1.17** | 27.25 | 0.2419219 | 0.2837933 | **0.60** | 0.048 |
| orf19.4055 | *YBR075W* | 388.0 | 714.1 | 431.1 | 285 | 505.9 | 233 | **1.17** | 18.87 | 0.4152538 | 0.3727254 |  |  |
| orf19.4146 | *SMD3* | 1000.0 | 2064.2 | 1247.1 | 666 | 1454.5 | 1013 | **1.17** | 0.309 | 0.6801266 | 0.2838693 | **0.77** | 0.050 |
| orf19.3159.4 | *UTP20* | 340.5 | 640.7 | 339.1 | 232 | 405.8 | 242 | **1.18** | 6.065 | 0.4524584 | 0.4452621 | **0.36** | 0.017 |
| orf19.4230 | *PRE4* | 698.4 | 1323.7 | 855.3 | 387 | 1008.8 | 739 | **1.18** | 0.359 | 0.8788984 | 0.722134 | **0.58** | 0.040 |
| orf19.3215 |  | 380.7 | 680.3 | 386.8 | 223 | 465.9 | 129 | **1.2** | 0.912 | 0.5215487 | 0.596158 | **0.56** | 0.016 |
| orf19.3939 |  | 338.8 | 582.8 | 352.6 | 262 | 422.9 | 312 | **1.2** | 4.397 | 0.459873 | 0.3539886 | **0.63** | 0.050 |
| orf19.1940 | *CAT5* | 780.3 | 1602.3 | 926.4 | 261 | 1122.6 | 456 | **1.21** | 0.307 | 0.407568 | 0.2010492 | **0.57** | 0.087 |
| orf19.4247 |  | 515.7 | 972.1 | 594.8 | 318 | 718.0 | 468 | **1.21** | 0.525 | 0.5907239 | 0.3682465 | **0.54** | 0.030 |
| orf19.972 |  | 275.5 | 470.5 | 257.1 | 148 | 318.3 | 114 | **1.24** | 3.093 | 0.3457812 | 0.4252267 | **0.68** | 0.097 |
| orf19.1107 |  | 750.3 | 1519.7 | 1006.8 | 970 | 1276.0 | 1358 | **1.27** | 0.753 | 0.8369047 | 0.6618112 | **0.63** | 0.036 |
| orf19.3163 |  | 362.4 | 696.8 | 372.1 | 169 | 472.8 | 177 | **1.27** | 1.099 | 0.2868334 | 0.3288561 | **0.67** | 0.063 |
| orf19.1123 |  | 248.2 | 457.3 | 238.1 | 212 | 308.4 | 92 | **1.29** | 69.46 | 0.4600485 | 0.4857858 | **0.61** | 0.028 |
| orf19.3169 |  | 1006.1 | 2184.2 | 1335.4 | 942 | 1760.1 | 1554 | **1.32** | 0.4 | 0.7504422 | 0.3518716 | **0.66** | 0.050 |
| orf19.5686 |  | 3217.5 | 8017.7 | 4673.9 | 4073 | 6290.3 | 5414 | **1.35** | 0.433 | 0.6626639 | 0.2137123 | **0.57** | 0.030 |
| orf19.3195 | *GAP3* | 1458.4 | 3502.7 | 1992.3 | 1414 | 2739.4 | 2178 | **1.37** | 0.465 | 0.4976186 | 0.0929755 | **0.58** | 0.034 |
| orf19.3932 |  | 2841.2 | 6996.7 | 3692.9 | 1313 | 5061.2 | 3582 | **1.37** | 0.418 | 0.3914844 | 0.1387116 | **0.61** | 0.064 |
| orf19.1927 |  | 418.2 | 910.9 | 438.1 | 153 | 617.4 | 143 | **1.41** | 0.801 | 0.2446252 | 0.3677452 | **0.60** | 0.032 |
| orf19.1117 | *FDH3* | 706.1 | 1584.8 | 891.0 | 599 | 1264.0 | 1101 | **1.42** | 0.595 | 0.5449368 | 0.2750555 | **0.74** | 0.060 |
| orf19.1953 |  | 415.6 | 911.6 | 445.5 | 233 | 645.7 | 283 | **1.45** | 1.037 | 0.3910008 | 0.4080582 | **0.60** | 0.043 |
| orf19.4220 |  | 477.6 | 1217.5 | 534.3 | 239 | 828.3 | 202 | **1.55** | 1.313 | 0.2515221 | 0.3223388 | **0.54** | 0.018 |
| orf19.4304 | *GAP1* | 378.2 | 900.5 | 412.7 | 240 | 673.2 | 611 | **1.63** | 0.957 | 0.3090194 | 0.1492443 | **0.62** | 0.053 |
| orf19.3934 | *CAR1* | 1032.6 | 2617.4 | 1306.6 | 543 | 2148.2 | 2011 | **1.64** | 0.832 | 0.6120703 | 0.3935858 | **0.60** | 0.038 |
| orf19.6659 | *GAP5* | 2133.9 | 6294.1 | 2985.3 | 2215 | 4951.1 | 4214 | **1.66** | 0.325 | 0.3566107 | 0.0074511 | **0.61** | 0.047 |
| orf19.3923 | *PGA37* | 836.8 | 2706.7 | 1155.5 | 1271 | 2426.6 | 2993 | **2.1** | 0.997 | 0.4555832 | 0.0969218 | **0.60** | 0.050 |
| orf19.4015 | *CAG1* | 256.9 | 1413.1 | 245.0 | 196 | 965.8 | 327 | **3.94** | 67.36 | 0.0402253 | 0.033765 | **0.64** | 0.047 |
| ^a^ Expression array values were normalized for all non-Ch5 genes. Background noise was subtracted. | | | | | | | | |  |  |  |  |  |
|  |  |  |  |  |  |  |  |  |  |  |  |  |  |
| ^b^ Genes expressed within the background noise on Ch5b in Sor55. | | | | | |  |  |  |  |  |  |  |  |
|  |  |  |  |  |  |  |  |  |  |  |  |  |  |
| Empty space for a CGH ratios corresponds to six genes that are absent on aCGH chips | | | | | | | |  |  |  |  |  |  |
