# Supplementary material for: Widespread Occurrence of Dosage Compensation in Candida albicans
Source: PLoS One. 2010 Jun 11;5(6):e10856. doi: 10.1371/journal.pone.0010856 (PMC2883996; doi:10.1371/journal.pone.0010856)
Supplement: Table S3 — RT-PCR validationa of the expression array values with 30 genesb on Ch5b in Sor55. (0.05 MB DOC) [file pone.0010856.s005.doc]

**Table S3**. RT-PCR validation^a^ of the expression array values with 30 genes^b^ on Ch5b in Sor55.

|  | | **Sor55/3153A, Ch5b** | |
| --- | --- | --- | --- |
| **Expression on Ch5b** | **Gene** | **Arrays** | **RT-PCR** |
| 4-fold up | *CAG1* | 3.9 | 3.9; 4.0; 4.2 |
| 2-fold up | *PGA37* | 2.1 | 2.0; 2.0; 2.7 |
|  | *GAP5* | 1.7 | 1.4; 1.5; 1.8 |
|  | *CAR1* | 1.6 | 1.3; 1.8; 1.8 |
|  | *GAP1* | 1.6 | 1.4; 1.6; 1.7 |
|  | orf19.4220 | 1.6 | 1.3; 1.4; 1.4 |
| Slightly up | orf19.5686 | 1.3 | 1.3; 1.4; 1.6 |
|  | *TEF4* | 1.2 | 1.0; 1.2; 1.3 |
| Disomic level | *HIS1* | 1.0 | 1.0; 1.0; 1.0; 1.1 |
|  | *UBC13* | 1.0 | 1.0; 1.0; 1.0 |
|  | *ACH1* | 1.0 | 1.0; 1.0; 1.0; 1.1 |
|  | *MDJ1* | 1.0 | 1.0; 1.0; 1.0 |
|  | *GLR1* | 1.0 | 0.8; 0.9; 0.9; 1.0; 1.1 |
|  | *PRE1* | 1.0 | 0.8; 0.9; 0.9; 0.9 |
|  | RPO26 | 1.0 | 0.9; 0.9; 1.0 |
|  | orf19.4248 | 1.0 | 0.8; 0.9; 1.0 |
|  | orf19.4349 | 1.0 | 0.8; 0.9; 1.0 |
|  | *CTA24*^c^ | 1.0 | 1.0; 1.0; 1.0; 1.2 |
| Intermediate & monosomic level | *SAH1* | 0.7 | 0.7; 0.7; 0.8 |
|  | *COR1* | 0.7 | 0.7; 0.7; 0. |
|  | *YNK1* | 0.6 | 0.4; 0.6; 0.6 |
|  | *VIP1* | 0.6 | 0.6; 0.7; 0.7 |
|  | *SEC14* | 0.5 | 0.5; 0.5; 0.5 |
|  | *THS1* | 0.5 | 0.5; 0.6; 0.7 |
|  | orf19.3216 | 0.5 | 0.5; 0.6; 0.6 |
|  | *PUT1* | 0.5 | 0.3; 0.5; 0.6 |
|  | *URA4* | 0.5 | 0.5; 0.6; 0.6 |
|  | *GDS1* | 0.5 | 0.2; 0.5; 0.6 |
| Disagreement with microarray | *SUI1* | 1.0 | 0.6; 0.7; 0.7; 0.7 |
|  | *SCW1* | 0.5 | 0.9; 0.8 |

^a^ See Fig. S1 for examples of semi-quantitative RT-PCR gels.

^b^ See Table S1 for gene systematic names.

^c^ Note that *CTA24* is duplicated on Ch5b in Sor55, Table S2 and legends to Fig. 5, and is serving here, as a control for the expression from two copies of gene. The other genes have aCGH ratios Sor55/3153A ranging from 0.51 to 0.68 and are, thus interpreted as represented by a single copy of the gene.

Transcript amounts for 30 genes on monosomic Ch5b in the mutant Sor55 were determined by semi-quantitative RT-PCR method. Data were obtained with RNA that was extracted from independently grown cultures using different or same control genes. Expression changes on Ch5b were calculated as mean ratios Sor55/3153A. Note the overall good agreement between array and RT-PCR values, except for *SCW11* and *SUI1*.
