# Supplementary material for: Widespread Occurrence of Dosage Compensation in Candida albicans
Source: PLoS One. 2010 Jun 11;5(6):e10856. doi: 10.1371/journal.pone.0010856 (PMC2883996; doi:10.1371/journal.pone.0010856)
Supplement: Table S4 — Northern blot validation of the array expression values with 18 genesa on Ch5b in Sor55. (0.03 MB DOC) [file pone.0010856.s006.doc]

**Table S4**. Northern blot validation of the array expression values with 18

genes^a^ on Ch5b in Sor55.

|  | | **Sor55/3153A, Ch5b** | |
| --- | --- | --- | --- |
| **Expression on Ch5b** | **Gene** | **Array** | **Northern analysis** |
| Slightly up | orf19.5686 | 1.3 | 1.0; 1.2; 1.3; 1.5 |
|  | *TEF4* | 1.2 | 0.7; 0.8; 0.8; 0.9 |
| Disomic level | *HIS1* | 1.0 | 0.9; 1.0; 1.1 |
|  | *CTA24*^b^ | 1.0 | 1.1; 1.2 |
|  | *PRE1* | 1.0 | 1.0; 1.1; 1.2; 1.4 |
|  | RPO26 | 1.0 | 0.7; 1.0 |
|  | orf19.4248 | 1.0 | 0.9; 1.0; 1.1; 1.2 |
|  | HSP122 | 1.0 | 0.8; 1.0; 1.0 |
| Intermediate & monosomic level | *SQT1* | 0.8 | 0.8; 1.2 |
|  | *RIB5* | 0.8 | 0.9; 1.1 |
|  | *SAH1* | 0.7 | 0.6; 0.8; 0.8; 1.0 |
|  | *COR1* | 0.7 | 0.5; 0.7; 0.7; 0.9 |
|  | *ERG11* | 0.6 | 0.8; 0.8 |
|  | *INT1* | 0.6 | 0.9; 0.9 |
|  | *ECM331* | 0.6 | 0.9; 1.0; 1.1 |
|  | *RPS11A* | 0.6 | 0.9; 1.0 |
|  | *ADH1* | 0.6 | 0.7; 0.9; 0.9; 1.0 |
|  | *FAS1* | 0.6 | 0.7; 0.8; 0.9; 0.9 |

^a^ See Table S1 for the gene systematic names.

^b^ *CTA24* is duplicated on Ch5b in Sor55 (see Results).

Data for each gene were obtained with RNA that was extracted from 2, 3 or 4 independently grown cultures using different or same control genes. Expression changes on Ch5b were calculated as mean ratios Sor55/3153A for each gene. Note the overall good agreement between array and Northern analysis values for the normalized or slightly up regulated, but not all down regulated genes.
